# Understanding the thermodynamic properties of insect swarms

**DOI:** 10.1038/s41598-021-94582-x

**Published:** 2021-07-22

**Authors:** Andy M. Reynolds

**Affiliations:** Rothamsted Research, Harpenden, Hertfordshire, AL5 2JQ UK

**Keywords:** Biophysics, Physics

## Abstract

Sinhuber et al. (Sci Rep 11:3773, 2021) formulated an equation of state for laboratory swarms of the non-biting midge *Chironomus riparius* that holds true when the swarms are driven through thermodynamic cycles by the application external perturbations. The findings are significant because they demonstrate the surprising efficacy of classical equilibrium thermodynamics for quantitatively characterizing and predicting collective behaviour in biology. Nonetheless, the equation of state obtained by Sinhuber et al. (2021) is anomalous, lacking a physical analogue, making its’ interpretation problematic. Moreover, the dynamical processes underlying the thermodynamic cycling were not identified. Here I show that insect swarms are equally well represented as van der Waals gases and I attribute the possibility of thermodynamic cycling to insect swarms consisting of several overlapping sublayers. This brings about a profound change in the understanding of laboratory swarms which until now have been regarded as consisting of non-interacting individuals and lacking any internal structure. I show how the effective interactions can be attributed to the swarms’ internal structure, the external perturbations and to the presence of intrinsic noise. I thereby show that intrinsic noise which is known to be crucial for the emergence of the macroscopic mechanical properties of insect swarms is also crucial for the emergence of their thermodynamic properties as encapsulated by their equation of state.

## Introduction

Collective behaviour in flocks, herds, crowds and swarms occurs throughout the biological world. Calling groups ‘collective’ suggests that the group is somehow more than, or at least different from, the sum of its individuals^1^. That is, care must be exercised to distinguish between emergent properties of the group that have no meaning at the individual level and aggregate properties that are simple averages over the participants. This is the approach taken in material science and in thermodynamics where response functions like viscosity or elastic moduli and state variables such as pressure and temperature can be defined and related to bulk materials without direct appeal to a molecular description. Ouellette^2,3^ championed applying such ideas to collective behaviour in biology. Previous studies have demonstrated that collective groups can have emergent material-like properties^4–7^ but Sinhuber et al.^8^ were the first to demonstrate empirically that collective groups can be described thermodynamically in terms of conventional state variables and their natural analogues. The most fundamental relationship for achieving this is the equation of state, which links the state variables that describe the macroscopic properties of the system and encodes how they co-vary in response to environmental changes. Sinhuber et al.^8^ formulated the first such equation of state. They formulated an equation of state for laboratory swarms of the non-biting midge *Chironomus riparius* and showed that this equation of state holds true when the swarms are driven through thermodynamic cycles in pressure–volume space via the application of a suitable sequence of external perturbations.

These findings of Sinhuber et al.^8^ demonstrate the efficacy of classical equilibrium thermodynamics for quantitatively characterizing and predicting collective behaviour in biology. This efficacy is surprising given the absence of conservation laws or well-described physical interactions and given the cognitive processing or agency of individuals. The significance of this breakthrough was not lost on Sinhuber et al.^8^ who noted rightly that such an “equation of state […] gives a new way both of illuminating the purpose of collective behaviour, given that it encodes the nature of the collective state, and quantitatively distinguishing different kinds of animal groups that may have similar movement patterns but different functions”.

Nonetheless, the equation of state obtained by Sinhuber et al.^8^ is anomalous, lacking a physical analogue, making its’ interpretation problematic. Moreover, the dynamical processes underlying the thermodynamic cycling were not identified. Here I address these problems, providing explanatory mechanisms for both the equation of state and the thermodynamic cycling. I show that the standard equation of state for van der Waals gases is as effective as Sinhuber et al.’s^8^ equation of state at encoding the thermodynamic properties of insect swarms. I also show that the thermodynamic cycling can be attributed to swarms being stratified: consisting of overlapping horizontal layers. Effective interactions and internal structure have until now escaped notice^9,10^. Finally, I suggest that because of their affinity with van der Waals gases, insect swarms can undergo liquid–gas phase transitions.

### Main contributions

- Provide the first evidence that swarms are stratified.
- Demonstrate that stratification together with the presence of intrinsic noise accounts for the thermodynamic properties of swarms as observed in the laboratory.
- Demonstrate similitude with van der Waals gas-like behaviour.
- Link results to acoustic interactions which are known to occur in insect swarms.

### In supplementary material

- Provide strong evidence that individuals are on average very weakly coupled inside swarms. This is a precursor for understanding the thermodynamic properties of swarms.
- Posit mechanisms for stratification.
- Demonstrate that swarms are critically damped and auxetic.

## Results

### No evidence for long-range interactions in laboratory insect swarms

Puckett et al.^10^ reported that acceleration measurements of laboratory swarms of the midge *Chironomus riparius* show a clear short-range repulsion but no conclusive evidence of long-range interactions between individuals and their nearest neighbours. In the Supplementary Material I I go beyond the pairwise analysis of Puckett et al.^10^ to examine the statistical properties of tetrahedra with vertices located at the positions of individuals and their 3 nearest neighbours. Tetrahedra are minimum characterizations of the spatial arrangement of individuals in 3 dimensions (since 3 individuals will always lie in a plane). I show that the statistical properties of the tetrahedra mirror expectations for Gaussian, independent individual positions, thereby bolstering the analysis of Puckett et al.^10^. Confirming the absence of long-range interactions in laboratory swarms is crucial for appropriately interpreting equations of state, which as shown later, are indicative of van der Waals gas-like behaviour. The apparent discrepancy is resolved after noting that perturbations (i.e., thermodynamic cycling) can induce correlations^11^ and herein) and after noting that equations of state are not swarm models per se, in that they do not make any detailed predictions about the dynamics of individuals. Rather, they give us a quantitative way of analysing and interpreting swarm data at the *macroscale*.

### ‘Slab confinement’

#### Observations

In this section I present evidence for laboratory swarms of the midge *Chironomus riparius* having internal structure. Evidence comes from 3 independent analyses that yield consistent results. In the first analysis I show that the vertical density profiles are accurately represented by superpositions of Gaussian density profiles, hereafter called ‘slabs’, and I show that the number of slabs in the fitted profiles increases with the average population size. This is, of course, not conclusive because any density profile can be represented by superpositions of Gaussian density profiles. Nonetheless, the decomposition into Gaussian density profiles is justifiable and physically plausible for insect swarms because they are a consequence of the gravitational-like restorative forces that are known to operate within insect swarms^9,12,13^. Moreover, the increase in the number of slabs with increasing population average population is indicative of a systematic change in the overall of shape (i.e., internal structure) of the swarm rather than with an increase in the overall size of the swarm. In the second analysis I show that mean acceleration profiles (i.e., mean restorative forces) are consistent with several centres of attraction each of which is expected to result in a local Gaussian density profile. In the third analysis I show that acceleration statistics can be accurately represented by superpositions of Gaussians. Further evidence for the swarms having internal structure comes from an unpublished study [van der Vaart, Private Communications documented herein] and from supporting analysis that is presented in the Supplementary Material II. Later it is shown that the identification of internal structure like the absence of correlations (documented above and in Supplementary Material I) is a crucial precursor for correctly interpreting equations of state and for understanding how the swarms can be driven through thermodynamic cycles by applying a suitable sequence of external perturbations. I show that observations cannot be replicated by simulations of swarms lacking internal structure.

The analysis draws inspiration from the observations of van der Vaart [Private Communication] who reported that smaller laboratory swarms are stratified: “They contain around 3 horizontal layers, with the lowest layer being flatter than the others. For larger laboratory swarms, individuals tend to explore a sub-part of the swarm, but the vertical centres of individual trajectories are distributed more homogeneously”. This internal structure was determined by visual expectation of the spatial extent of long trajectories which because of tracking errors are rarely recorded. Here in the first of 3 analyses slab confinement is determined objectively and directly from density profiles computed from all recorded trajectories, using a standard, robust statistical method.

**Figure 1:**
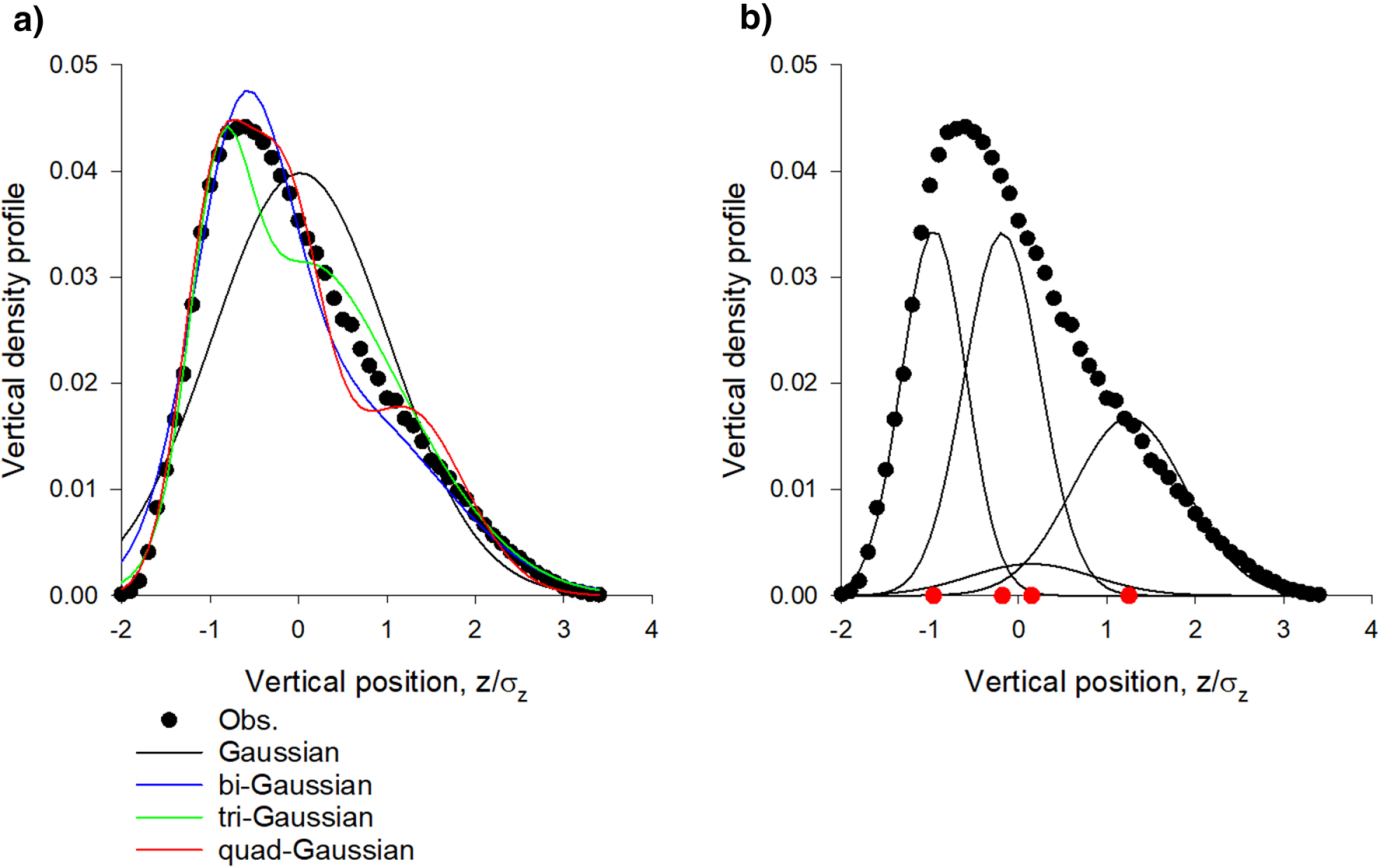
(**a**) Vertical density profiles of laboratory swarms are accurately represented by superpositions of Gaussians. (**b**) Constituent Gaussians in the quad-Gaussian representation with centres at •. In accordance with observations, the best superposition **“**contains around 3 horizontal layers, with the lowest layer being flattered than the others” [van der Vaart, Private Communication]. The Akaike weights for the single-, bi-, tri- and quad-Gaussian fits are 0.00, 0.00, 0.08 and 0.92 indicating strong support for the quad-Gaussian representation (albeit with one weak ‘embryonic’ slab). Data are taken from Sinhuber et al.^14^: swarm Ob1 which contains on average 94 individuals. The swarm is centred on z = 0.

**Figure 2:**
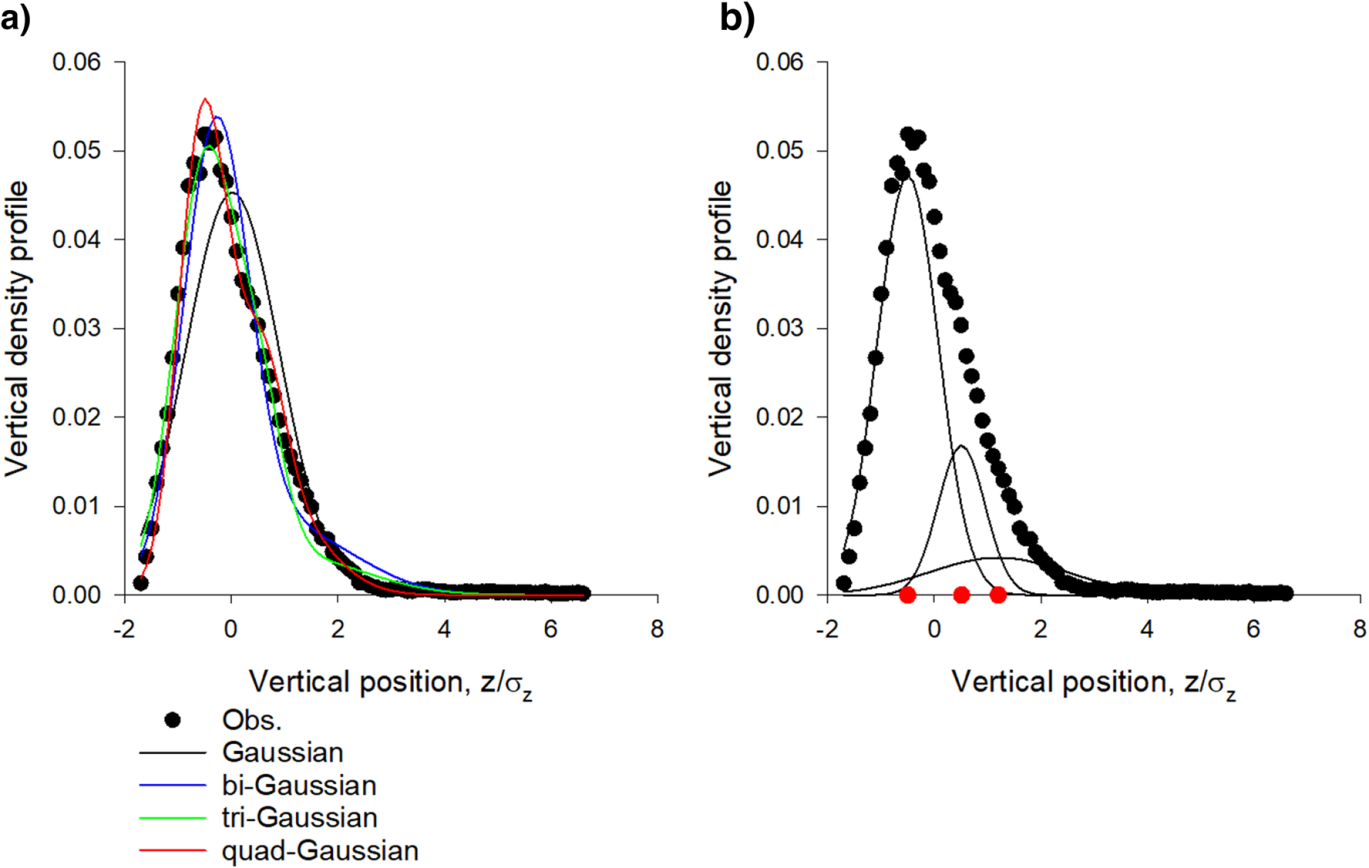
(**a**). Vertical density profiles of laboratory swarms are accurately represented by superpositions of Gaussians. (**b**) Constituent Gaussians in the tri-Gaussian representation with centres at •. In accordance with observations, the best superpositions **“**contains around 3 horizontal layers, with the lowest layer being flattered than the others” [van der Vaart, Private Communication]. The Akaike weights for the single-, bi-, tri- and quad-Gaussian fits are 0.00, 0.78, 0.21 and 0.01 indicating strong support for the bi and tri-Gaussian representations. Data are taken from Sinhuber et al.^14^: swarm Ob5 which contains on average 22 individuals. The swarm is centred on z = 0.

Multi-Gaussian density profiles,

1 $$ p\left( z \right) = \mathop \sum \limits_{i = 1}^{N} w_{i} \frac{{exp\left( { - \frac{{\left( {z - \overline{{z_{i} }} } \right)}}{{2\sigma_{i}^{2} }}} \right)}}{{\sqrt {2\pi } \sigma_{i} }} $$

with $\sum\nolimits_{i = 1}^{W} {w_{i} = 1}$ where fitted to observed vertical density profiles using maximum likelihood estimation methods and the best model profile was determined using the Akaike criterion. The Akaike weights, which range between 0 and 1, are the weights of evidence in favour of the Nth-order multi-Gaussian being the best representation for the given data, out of the multi-Gaussians distributed considered. Here N = 1, 2, 3 and 4 are considered. All 17 dusk time swarms in the dataset of Sinhuber et al.^14^ provide strong support for the presence of slabs. The best fit distributions mirror the observations of van der Vaart (Figs. 1, 2). Comparable fits are obtained for the 1st and 2nd halves of the recordings, indicating that the slab structure does not change significantly over time. Density profiles in the horizontal directions, on the hand other, are closely Gaussian.

**Figure 3:**
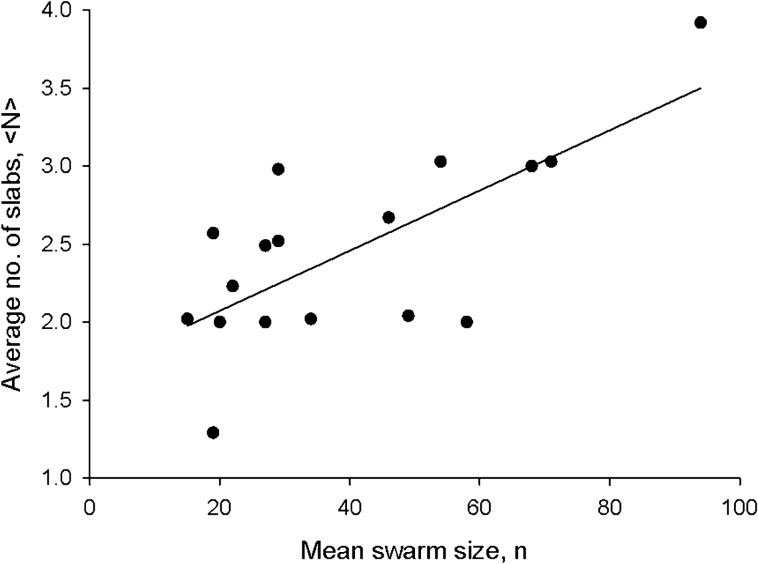
Average number of Gaussian slabs $N = \sum\nolimits_{N = 1}^{4} {w_{N} N}$ tends to increase with increasing average population size, n. w_N_ are the Akaike weights for best-fit profile containing N slabs. The least-squares linear regression (solid line) is added to guide the eye (R^2^ = 0.50). Data are taken from Sinhuber et al.^14^. All 17 daytime swarms.

The number of slabs tends to increase with increasing average population size (Fig. 3, also compare Figs. 1, 2). This remains the case when the swarms are sub-sampled so that the number of positional fixes in the analyses remains constant irrespective of the average population size. The increase in the number of slabs with increasing average population cannot therefore be attributed to it becoming statistically more favourable to include more components but can instead to attributed to a systematic change in the overall shape of the swarm with increasing average population, as swarm growth proceeds slab by slab (this is not true generally of arbitrarily defined density profiles, Supplementary Material III). This suggests in accordance with observations that very large swarms will be cylindrical^15^.

**Figure 4:**
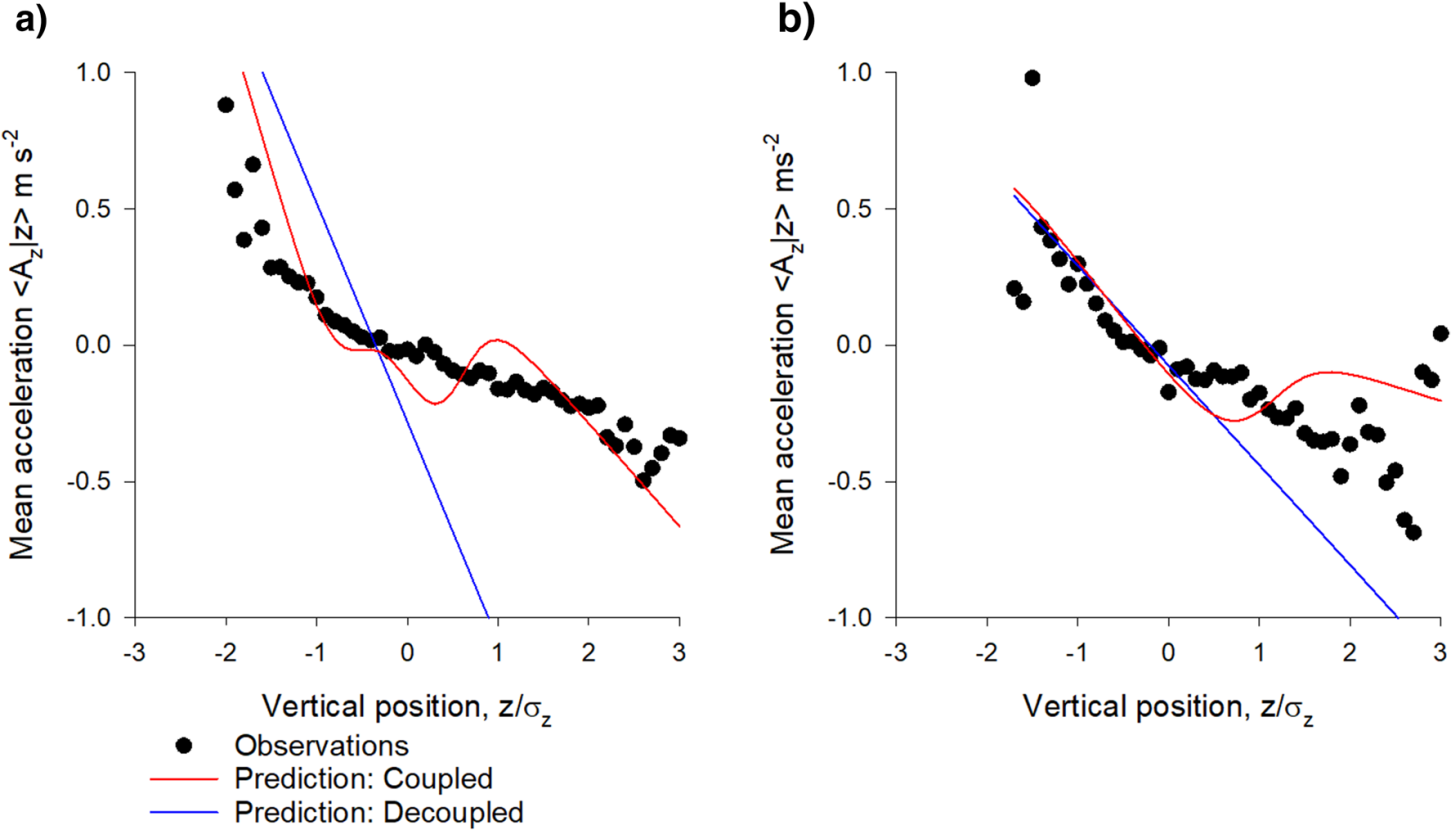
Mean accelerations are consistent with slabs being coupled. Data (•) are shown (**a**) for swarm (Ob1) which contain on average 94 individuals and (**b**) for a swarm (Ob5) which contains on average 22 individuals. Shown for comparison are model predictions for coupled slabs, Eq. (2) (red lines) and for decoupled slabs, Eq. (3) (blue lines). Data are taken from Sinhuber et al.^14^.

Stochastic models for the simulation of individual swarming insects are in close agreement with all available data^7,11,16–18^. Such modelling indicates that individuals occasionally move between slabs, i.e., that the slabs are coupled rather than decoupled. The modelling approach predicts that mean vertical accelerations of individual insects are given by

2 $$ A_{z} {|}z = - \frac{{\sigma_{w}^{2} }}{P\left( z \right)}\mathop \sum \limits_{i = 1}^{N} w_{i} \frac{{\left( {z - \overline{{z_{i} }} } \right)}}{{\sigma_{i}^{2} }}\frac{{exp\left( { - \frac{{\left( {z - \overline{{z_{i} }} } \right)}}{{2\sigma_{i}^{2} }}} \right)}}{{\sqrt {2\pi } \sigma_{i} }} $$

when the slabs are coupled and by

3 $$ A_{z} {|}z = - \sigma_{w}^{2} \mathop \sum \limits_{i = 1}^{N} w_{i} \frac{{\left( {z - \overline{{z_{i} }} } \right)}}{{\sigma_{i}^{2} }} $$

when the slabs are decoupled, where $\sigma_{w}^{2}$ is the velocity variance. The former unlike the latter provides a good fit to data (Fig. 4). Nonetheless, nonmonotonicity of the mean accelerations predicted by this simple coupling does not have experimental support. Better agreement with experiment may be obtained with a weaker form of coupling that lies between Eq. (2) and the monotonic form given by Eq. (3).

This goodness of fit provides further evidence for slab confinement; slab confinement accounts for the departures from a linear force law (harmonic potential) that operates in the vertical direction but not in the horizontal directions. This suggests that the mean acceleration (effective restorative force) depends on the probability densities. In the outskirts of swarms, where densities are low, effective restorative forces are purely harmonic (gravitational-like, Okubo^13^ Fig. 4). In the cores of swarms with overlapping sublayers, where densities are high, the effective forces are reduced relative to expectations for slab-less swarms (Fig. 4). This is closely akin to so-called ‘adaptive gravity’ models of insect swarms^12^. In these models, the strength of the interactions between individuals is density dependent. This dependency is crucial for bringing adaptive gravity models into agreement with observations of both the average forces within the swarms and an analogue of the virial theorem based on mass conservation.

**Figure 5:**
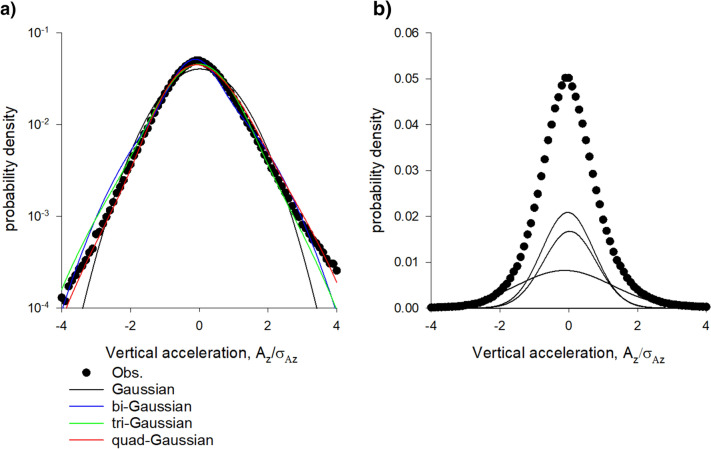
(**a**). Distributions of vertical accelerations are accurately represented by superpositions of Gaussians. (**b**) Constituent Gaussians in the tri-Gaussian representation with centres at •. The Akaike weights for the single-, bi-, tri- and quad-Gaussian fits are 0.00, 0.03, 0.89 and 0.09 indicating strong support for the tri-Gaussian representation. Data are taken from Sinhuber et al.^14^: swarm Ob1 which contains on average 94 individuals.

**Figure 6:**
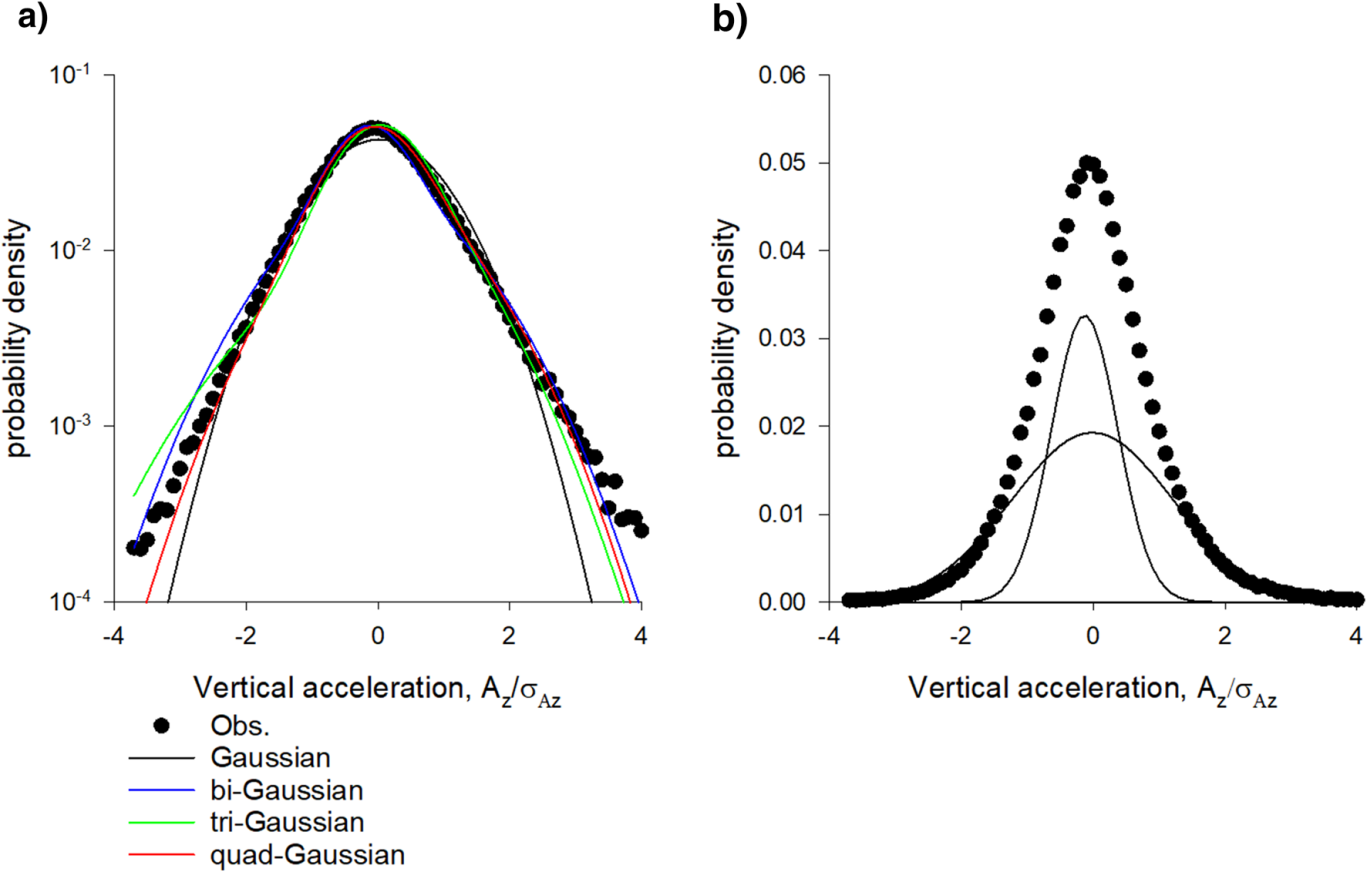
(**a**). Distributions of vertical accelerations are accurately represented by superpositions of Gaussians. (**b**) Constituent Gaussians in the tri-Gaussian representation with centres at •. The Akaike weights for the single-, bi-, tri- and quad-Gaussian fits are 0.05, 0.79, 0.14 and 0.02 indicating strong support for the bi-Gaussian representation. Data are taken from Sinhuber et al.^14^: swarm Ob5 which contains on average 22 individuals.

Distributions of acceleration, like density profiles, can be accurately represented by multi-Gaussians (Figs. 5, 6). The number of Gaussians typically matches the number the Gaussian representations of the density profiles, i.e., the number of slabs and the number of flight modes are typically the same. By way of contrast, velocities are closely Gaussian, as noted by Kelley and Ouellette^9^.

#### Emergent mechanical-like properties of slabs can be attributed to intrinsic noise

The vertical size of the slabs tends to increase with height. This is consistent with the observations of slab confinement made by van der Kaart [Private Communication] and with observations of Kelley and Ouellette^9^ who reported that laboratory swarms tend to have diffuse tops. From an emergent mechanical-properties perspective, the lower slabs are being compressed by the slabs above them. Here I show that this emergent property, like the emergence of tensile strength (Reynolds 2020), can be attributed to the presence of intrinsic noise. [Note that in the absence of sublayers, intrinsic (multiplicative) noise causes isolated swarms to expand (Reynolds 2020)]. In the Supplementary Material IV I show how slab formation can be attributed to intrinsic noise and I suggest that swarms are poised at the cusps of phase transitions (i.e., are close to spontaneously creating new slabs which will change a swarm’s internal structure).

**Figure 7:**
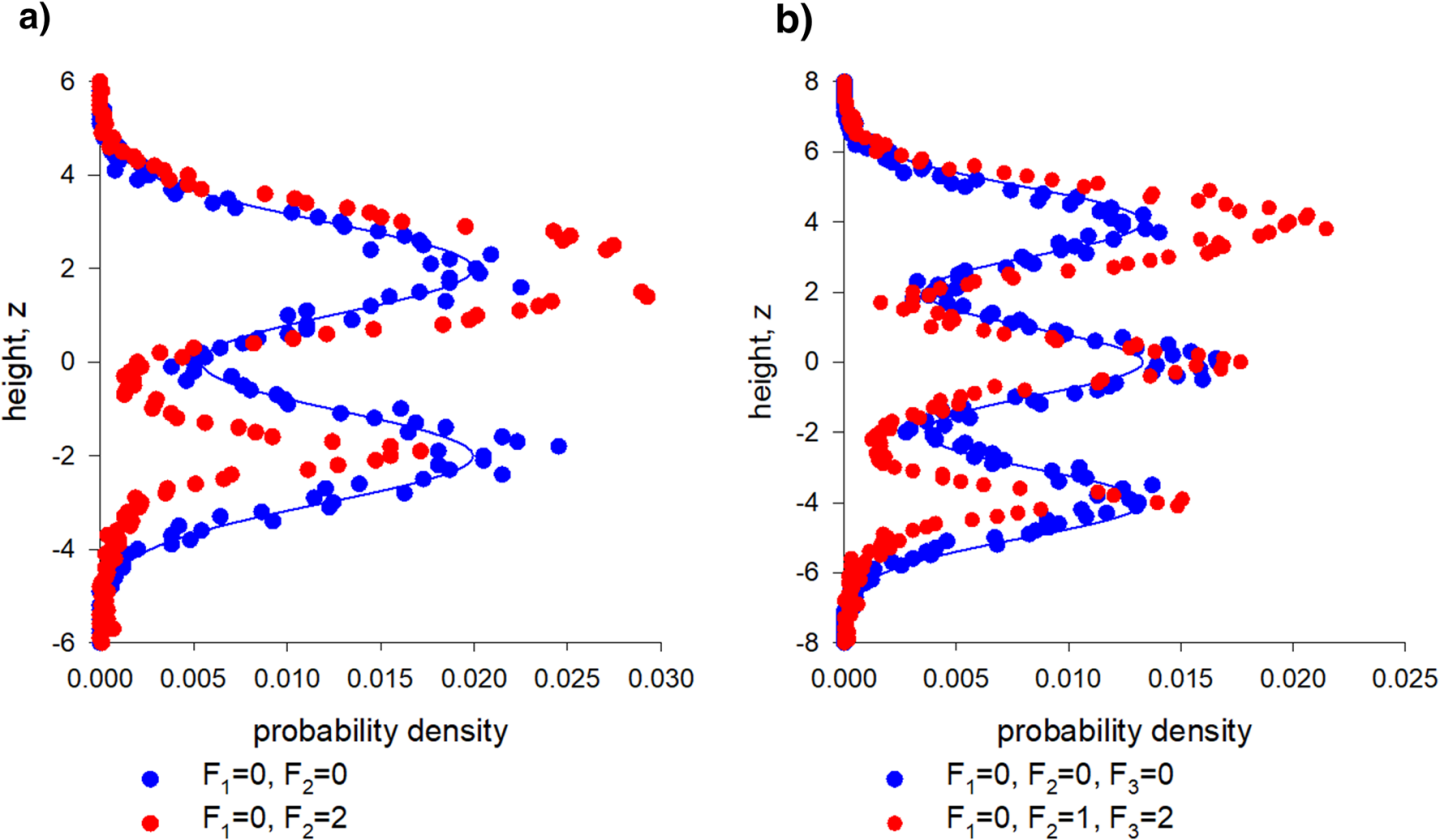
Intrinsic noise causes compression and de-population of lower sublayers. In the absence of intrinsic noise, distributions of simulated individuals (•) match theoretical expectations (solid line) for noiseless swarms. The presence of noise in the lower sub-layers reduces their size and causes partially de-populate. Predictions are shown for the stochastic model, Eq. (4) (**a**) and its generalization to 3 sublayers (**b**).

A simple random walk model for the vertical movements, *z*, of individuals within a swarm which in the absence of intrinsic noise consists of two Gaussian sublayers centred on $\overline{{z_{1} }}$ and on $\overline{{z_{2} }}$ with unit variance is given by

4 $$ \begin{aligned} dz & = U_{1} \left( {dt + F_{1} \sqrt 2 d\xi_{1} } \right) + U_{2} \left( {dt + F_{2} \sqrt 2 d\xi_{2} } \right) + F_{3} \sqrt 2 d\xi_{3} \\ U_{1} & = - \left( {z - \overline{{z_{1} }} } \right)exp\left( { - \frac{{\left( {z - \overline{{z_{1} }} } \right)^{2} }}{2}} \right) \\ U_{2} & = - \left( {z - \overline{{z_{2} }} } \right)exp\left( { - \frac{{\left( {z - \overline{{z_{2} }} } \right)^{2} }}{2}} \right) \\ \end{aligned} $$

as evidenced by the results of numerical simulations shown in Fig. 7; where $d\xi_{i} \left( t \right)$ are independent incremental Wiener process with correlation property $\overline{{d\xi_{i} \left( t \right)d\xi_{j} \left( {t + \tau } \right)}} = \delta \left( \tau \right)\delta_{ij} dt$ and where the constants *F*_*i*_ set the noise intensities (For details of model formulation see Supplementary Material V^17,19^. Noise in the lower sublayer results in compression and suppression (i.e., partial de-population) of that sublayer, as individuals migrate to the upper sublayer which expands and becomes more prominent. This could create strong feedback, as de-population is likely to be accommodated by increased intrinsic noise in the lower slab (fluctuations arise in part from the limited number of individuals in the sublayers). Note that intrinsic noise in the lower slabs could be relatively large because of the presence of the ground which disrupts individual trajectories. Note also that the presence of small multiplicative noise can dramatically shorten escape times from the potential wells^13^.

#### Dynamic response to perturbations

**Figure 8:**
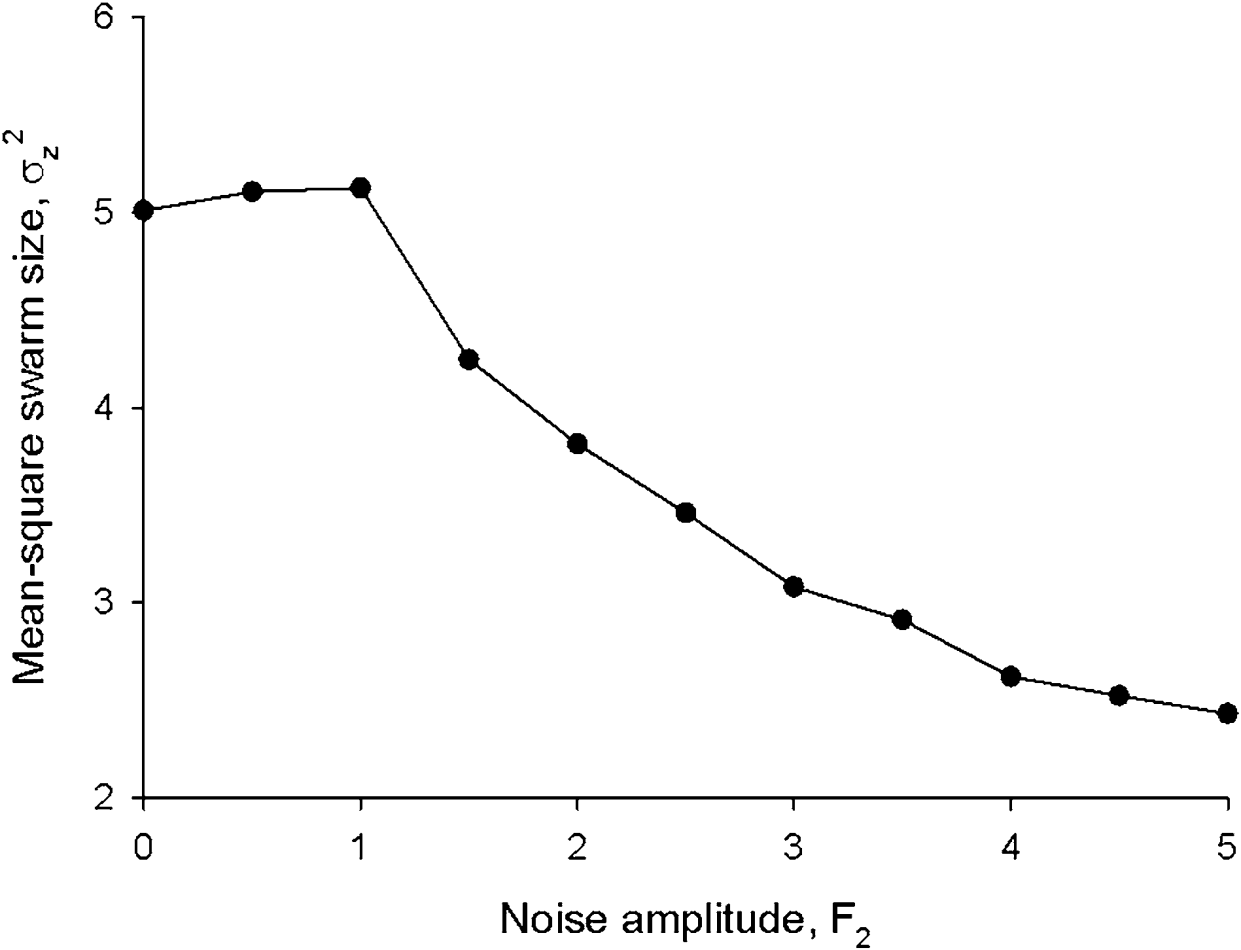
Swarms are predicted to compress in response to external dynamic perturbations. Predictions were obtained using the stochastic model, Eq. (4), for the bipartite swarm shown in Fig. 7a.

A key open question for understanding the properties of collective systems is how they respond to perturbations. Sinhuber et al.^20^ examined this with precisely controlled laboratory experiments. They considered the effect of a controlled variable light exposure on the swarming behaviours of the *Chironomus riparius* midge. They found that a swarm responds to these perturbations by compressing and simultaneously increasing the attraction of individual midges to its centre of mass. The compression can be understood using the above approach since parametric noise causes the slab structure to change (Fig. 7) thereby causing swarms to compress (Fig. 8). In this case the parametric noises, *F*_*1*_ and *F*_*2*_ have both intrinsic and external components. Compression of the swarm typically arises when the amplitude of the response to the external perturbation varies between slabs. This is reasonable given that slab formation was, here, linked to flight mode, i.e., to motor function (Figs. 5, 6). The reported increase in attraction appears to conflict with the model which predicts that the average restorative forces remain unchanged. Sinhuber et al.^20^ plotted the average restorative forces as a function of $\left( {z - \overline{z}} \right)/\sigma_{z}$ rather than as a function of z. The model predicts that these plots will change because both $\overline{z}$ and $\sigma_{z}$ are noise dependent; giving the impression that the attractive force has increased.

### Equations of state for insect swarms

#### Models

Sinhuber et al.^8^ demonstrated that insect swarms can be described in a thermodynamic framework; as championed by Ouellette^2,3^. Sinhuber et al.^8^ formulated an equation of state that holds true when the swarms are driven through thermodynamic cycles by the application visual and acoustic perturbations (light on, no noise; light on, noise; light off, noise, light off, noise off). Here I show that a closely analogous equation of state characterizes simulated swarms and that it holds true when through cycling through the strengths of noises (strong multiplicative noise, weak additive noise; strong multiplicative noise, strong additive noise; weak multiplicative noise, strong additive noise; weak multiplicative noise, weak additive noise). The predicted effects of the multiplicative noise are shown to resemble the observed effects of the light on/off perturbation which move the state of the swarm along an isotherm^14^ (see above) and the predicted effects of the additive noise are shown to resemble the observed effects of the acoustic on/off perturbation. The distinction arises because unlike additive noise, multiplicative noise can change the shape of a potential well^13^, (Reynolds 2020) (Fig. 7).

Sinhuber et al.^8^ identified the state variables to be: the volume of the swarm,an analogue of temperature that is proportional to the kinetic energy; pressure; and the number of individuals in the swarm. Here the state variables are taken to be: the swarm size, $\sigma_{z}$ (a proxy for volume); the velocity variance $\sigma_{w}^{2}$ (i.e., twice the kinetic energy, a proxy for temperature); and, as with Sinhuber^8^, pressure, $P = \left( {\sigma_{w}^{2} + zA} \right)/\sigma_{z}$ where, *z* is the vertical position relative to the swarm centre and *A* is the mean acceleration. The acceleration term is motivated by the fact that insects in a swarm behave as if they are trapped in a harmonic potential well^9,13^, and captures the work by an insect as it accelerates in the potential. Note that this global pressure is not representative of local pressures which differ in the core and in the outskirts of the swarm^1^, Supplementary Material VI). The number of individuals is not considered because it is constant in the simulated swarms. Following Sinhuber et al.^8^ an equation of state was found by assuming the functional form

5 $$ P = f\left( {\sigma_{z} ,\sigma_{w} } \right) = c_{1} \sigma_{z}^{{c_{2} }} \sigma_{w}^{{c_{3} }} $$

and using non-linear regression where *c*_*1*_, *c*_*2*_ and *c*_*3*_ are constants. This procedure requires a stochastic model for both the positions and velocities of individuals within a swarm, i.e., it requires that Eq. (4) be replaced by

6 $$ \begin{aligned} dw & = - wdt + U_{1} \left( {dt + F_{1} \sqrt 2 d\xi_{1} } \right) + U_{2} \left( {dt + F_{2} \sqrt 2 d\xi_{2} } \right) + \sqrt 2 d\xi_{3} \\ dz & = wdt \\ U_{1} & = - \left( {z - \overline{{z_{1} }} } \right)exp\left( { - \frac{{\left( {z - \overline{{z_{1} }} } \right)^{2} }}{2}} \right) \\ U_{2} & = - \left( {z - \overline{{z_{2} }} } \right)exp\left( { - \frac{{\left( {z - \overline{{z_{2} }} } \right)^{2} }}{2}} \right) \\ \end{aligned} $$

**Figure 9:**
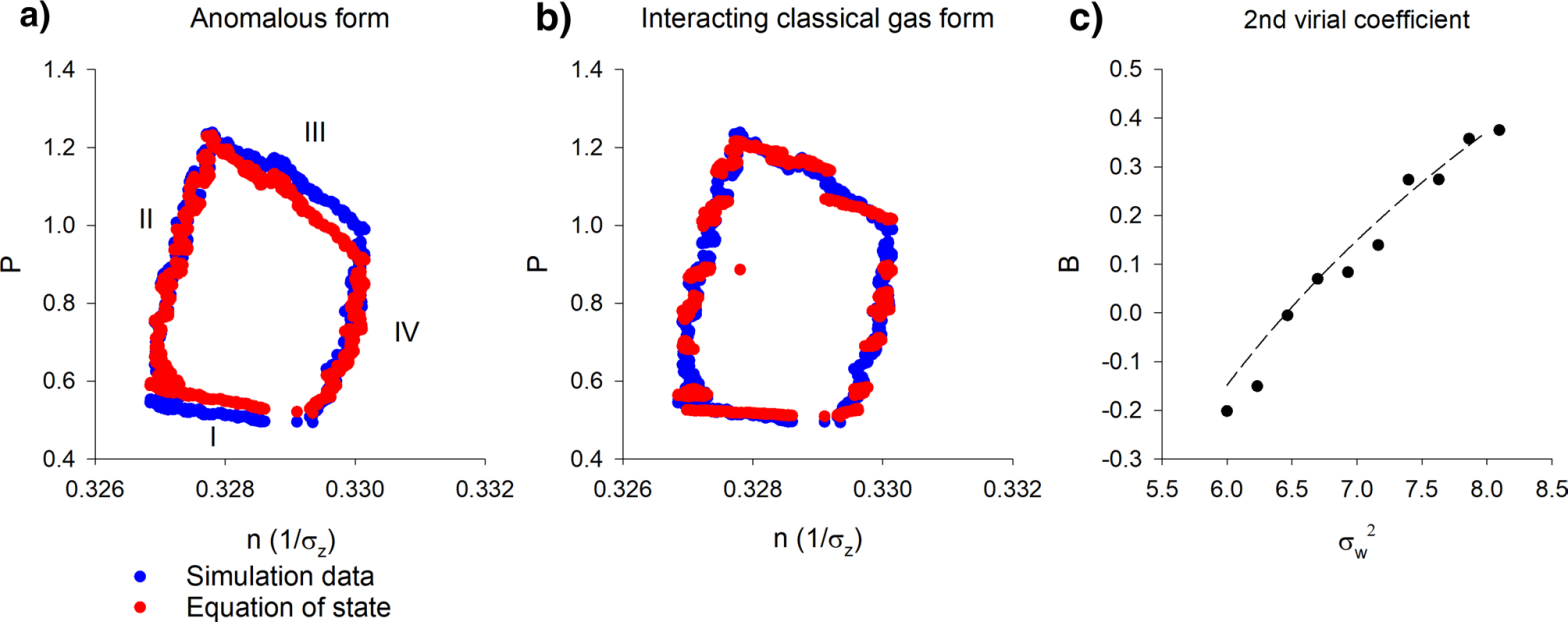
Simulated and predicted thermodynamic cycling. Simulation data were obtained using the simple stochastic model, Eq. (4), for the bipartite swarm shown in Fig. 7a. Cycling the strengths of noises (strong multiplicative noise, weak additive noise; strong multiplicative noise, strong additive noise; weak multiplicative noise, strong additive noise; weak multiplicative noise, weak additive noise) drives the simulated swarm through a ‘thermodynamic’ cycle that resembles observations^8^. (**a**) Predictions were obtained using the equation of state, Eq. (5), with c_1_ = 0.001, c_2_ = 1.59, c_3_ = 2.69 and (**b**) with the virial-like expansion. (**c**) The second-order virial coefficient was obtained from fits to the simulation data. The second-order virial coefficient is well represented by $B = a - \frac{b}{{\sigma_{w}^{2} }}$ (dashed-line) which corresponds to a van der Waals gas. Each phase has duration 0.25 (a.u.) and F_1_ = 1 throughout. Phase I F_2_ = 2, F_3_ = 1; Phase II F_2_ = 2, F_3_ = 2; Phase III F_2_ = 0, F_3_ = 2; Phase IV F_2_ = 0, F_3_ = 1.

[For details of model formulation see Supplementary Material V^17–19^]. When the simulated swarm is driven through a thermodynamic cycle (by cycling the additive and multiplicative noises), the equation of state, $P\sigma_{z}^{1.6} = 0.003\sigma_{w}^{2.8 }$ holds throughout (Fig. 9a). Sinhuber et al.^8^ reported that $PV^{1.7} \propto NT^{2}$. Observed and predicted collective behaviours are thus similar and surprisingly simple. Note, however, that the simulation data (over the available ranges) for pressure is equally well represented by the first two terms in the virial-like expansion, $P \propto \frac{{\sigma_{w}^{2} }}{{\sigma_{z} }}\left( {A + \frac{1}{{\sigma_{z} }}B\left( {\sigma_{w}^{2} } \right) + \frac{1}{{\sigma_{z}^{2} }}C\left( {\sigma_{w}^{2} } \right) \ldots } \right)$, for an interacting gas (Fig. 9b,c). The second-order virial coefficient is well represented by $B = a - \frac{b}{{\sigma_{w}^{2} }}$ (Fig. 9c) which corresponds to a van der Waals gas^21^. This does not indicate that individuals are interacting—simulated individuals are not interacting –nonetheless they are behaving as if they are interacting.

The apparent interactions can be attributed to the multiplicative noise, which is present, even in quiescent swarms^22^. Consider for simplicity a swarm without slabs. Stochastic modelling predicts that the joint distribution of positions and velocities is given by

7 $$ P\left( {z,w} \right) = \left( {1 + \frac{F}{{\sigma_{w}^{2} T_{c} }}z^{2} } \right)^{{ - \frac{{\sigma_{w}^{2} T_{c} }}{{2\sigma_{z}^{2} F}} - 1}} exp\left( { - \frac{{w^{2} }}{{2\sigma_{w}^{2} }}} \right)/Q $$

where *F* is the magnitude of the multiplicative noise, $\sigma_{z}$ is the root-mean-square size of the swarm in the absence of multiplicative noise, $\sigma_{w}$ is the root-mean-square speed of the individuals, *T*_*c*_ is a velocity-autocorrelation timescale and *Q* is a normalization factor, i.e., the canonical partition function^22^. In the absence of multiplicative noise, positions and velocities are jointly Gaussian. In this case, the canonical partition function

8 $$ lnQ = \frac{1}{2}ln\left( {2\pi \sigma_{w}^{2} } \right) + \frac{1}{2}ln\left( {2\pi \sigma_{z}^{2} } \right) $$

is directly analogous to that for a non-interacting gas,

$$ lnQ = \frac{1}{2}ln\left( {2\pi kT} \right) + ln\left( V \right) $$

But in the presence of multiplicative noise, the canonical partition function

9 $$ lnQ = \frac{1}{2}ln\left( {2\pi \sigma_{w}^{2} } \right) + \frac{1}{2}ln\left( {2\pi \sigma_{z}^{2} } \right) - \frac{F}{4}\frac{{\sigma_{z}^{2} }}{{\sigma_{w}^{2} }} + O\left( {F^{2} } \right) $$

This is directly analogous to the canonical partition function for an interacting gas

10 $$ lnQ = \frac{1}{2}ln\left( {2\pi kT} \right) + ln\left( V \right) - \frac{1}{2}VB\left( T \right) $$

where $B\left( T \right)$ is second virial coefficient, if $F = F_{0} /\sigma_{z}$. The simple ansatz for *F* is reasonable because the magnitude of the intrinsic noise is found to decrease as the size of the swarm increases^22^.

Intrinsic multiplicative noise may therefore account for both the mechanical properties of swarms—tensile strength, yield strength—and their thermodynamic properties—equations of state.

#### Heuristic understanding of the effective correlations

In the absence of slabs, multiplicative noise causes swarms to expand, as if the individuals are being repelled from one another, as indicated by B > 0. In the presence of slabs, multiplicative noise causes swarms to contract (Figs. 7, 8), as if the individuals are being attracted to another, as indicated by B < 0. This provides further evidence for the presence of slabs within insect swarms. Also note that at high temperatures (above the Boyle temperature where *B* = *0*), repulson dominates.

#### Observations

**Figure 10:**
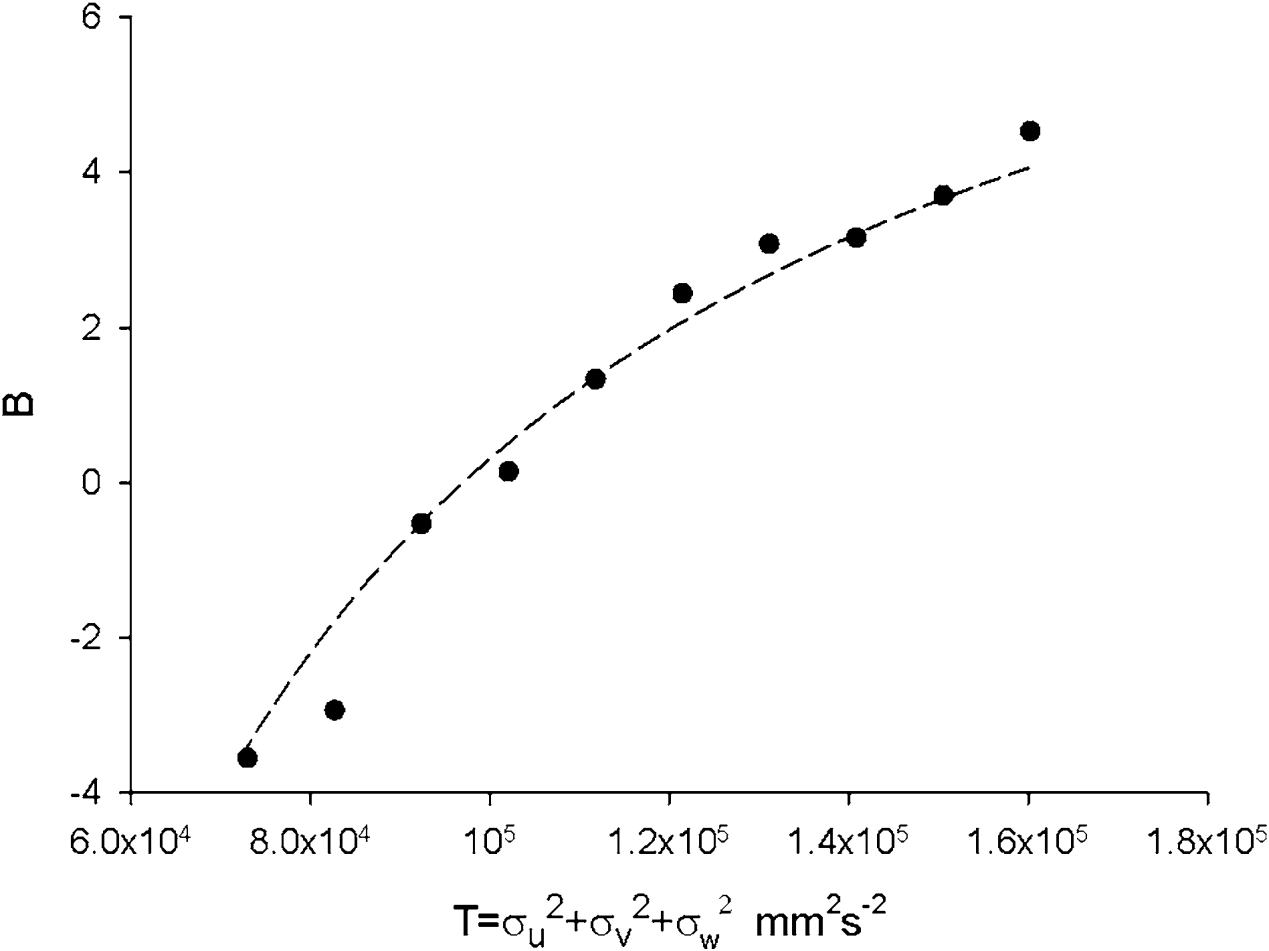
Second virial coefficient for the largest laboratory swarm (•) mirrors theoretical expectations for a swarm with slabs and corresponds to a van der Waals gas (dashed line). Data are taken from Sinhuber et al.^14^; swarm Ob1 which contains on average 94 individuals.

In accordance with Sinhuber et al.^8^ the largest laboratory swarm (Ob1) in the dataset of Sinhuber et al.^14^ containing on average 94 individuals was found to be well characterised by an equation of state *PV*^*1.7*^ = *kNT*^*2*^. As with the simulated swarms, it is equally well characterized by an equation of state for a non-ideal gas (Fig. 10). For the smaller laboratory swarms, second virial coefficients increase with increasing kinetic energy (temperature) and so resemble those for interacting gases, but they do not adhere closely to the van der Waals form. It is therefore possible that the equation of state attains an asymptotic form pertaining to a van der Waals gas when swarms contain order 10 individuals. This would be consistent with the observations of Puckett and Ouellette^23^ who reported that once the swarms contain around 10 individuals, average volume occupied per individual, average nearest-neighbour distance, along with other swarm statistics saturate.

#### Equipartition

In conventional statistical mechanics, the equipartition theorem states that a classical system in thermal equilibrium at temperature *T* has a mean energy of *kT*/2 per degree of freedom where *k* is a constant. Sinhuber et al.^8^ showed that equipartition holds for the laboratory swarms despite not being in thermal equilibrium and that the number of degrees of freedom is about 6: 3 translational and 3 potential which not surprising given that potential well in which the insects reside is 3-dimensional^9^. It is readily shown that equipartition also holds for the stochastic swarm models^17,19^ (Reynolds et al. 2017). In one-dimensional versions of these models for statistical stationary swarms, the mean acceleration (restorative force) is given by

11 $$ F\left( {u,x} \right) = - \frac{1}{{P\left( {u,x} \right)}}\frac{\partial }{\partial x}\mathop \smallint \limits_{ - \infty }^{u} vP\left( {v,x} \right)dv $$

where $P\left( {u,x} \right)$ is the joint distribution of position and velocity^19^, Reynolds 2017). The potential energy

$$ E_{p} = \frac{1}{2}xF = - \mathop \smallint \limits_{ - \infty }^{\infty } \mathop \smallint \limits_{ - \infty }^{\infty } x\frac{\partial }{\partial x}\mathop \smallint \limits_{ - \infty }^{u} vP\left( {v,x} \right)dvdudx = \frac{1}{2}\sigma_{u}^{2} $$

is therefore equal to the kinetic energy, $E_{k} = \frac{1}{2}\sigma_{u}^{2}$. More elaborate analysis shows that this is also true of 2- and 3-dimensional stochastic models of quiescent swarms. This is a consequence of the virial state which for stationary systems dictates that $2E_{k} - 2E_{p} + S = 0$ where S = 0 is the surface pressure. Nonetheless, the results of numerical simulations show that in the presence of multiplicative noise, $E_{k} > E_{p}$; indicating that equipartition is not satisfied exactly and that S < 0 in accordance with observations^12^. This was anticipated by Reynolds^17,18,22^ who attributed S < 0 (i.e., confining surface pressures) to erratic movements of the swarms’ centre of mass.

## Discussion

The equation of state extracted by Sinhuber et al.^8^ quantifies the macroscopic properties of the swarms. Its’ nonlinear form shows that these macroscopic properties cannot be the result of individuals behaving like an ideal gas. This is unexpected because other studies indicate that individuals although tightly bound to the swarm itself are weakly coupled inside it^10^ and herein in Supplementary Material I]. Moreover, stochastic models of the trajectories of *non-interacting* midges agree with numerous observations from carefully controlled, high precision, laboratory experiments. They do, for example, account: for the observed emergence of macroscopic mechanical properties similar to solids, including a finite Young’s modulus and yield strength^4,17,18^; for the observed collective viscoelastic response to applied oscillatory visual stimuli^7^; and for the fact that laboratory swarms of the non-biting midge *Chironomus riparius* consist of a core ‘condensed’ phase surrounded by a dilute ‘vapour’ phase^1,17^.

Here such stochastic models were shown to account for the equation of state obtained by Sinhuber et al.^8^ but only when they take explicit account of slab confinement which was uncovered here (Figs. 1, 2, 3, 4) in an analysis of pre-existing data^20^. It was also shown how this internal structure allows swarms to be driven through thermodynamic cycles by the application of a suitable sequence of external perturbations to the swarms (Figs. 7, 8, 9). Departures of the effective equation of state from the ideal gas law were here attributed to the swarms’ internal structure and to presence of extrinsic and intrinsic noise rather than to interactions per se between individuals.

Here it was shown that predicted and observed departures from the ideal gas law are (over the accessible parameter ranges) equally well represented by the non-linear equation of state advocated by Sinhuber et al.^8^ which lacks a physical interpretation and by the conventional equations of state for van der Waals gases (Figs. 9c, 10). Both thermodynamic descriptions are succinct yet complete and accurate. The non-uniqueness is not surprising given that similar macroscopic properties can emerge from different microscopic dynamics, i.e., emergent macroscopic properties can be insensitive to microscopic details. Nonetheless, it was shown how similitude with van der Waals gases is readily understood, thereby providing new insights into swarming.

Slab confinement could be facilitated by acoustic ordering. It has been suggested that to best facilitate detection of females, male mosquitoes within swarms seek to reduce the acoustic interference they create for another by dividing the sounds they make into local clusters with unique tones^24^. Aldersley et al.^25^ subsequently revealed the formation of frequency-domain clusters between individuals within audible range of one another. They noted that this may be important for swarm formation and cohesion because flight tones are the interactive media through which inter-individual coupling and movement coordination takes place. Indeed, Reynolds^18^ showed that the cohesion of midge swarms can be attributed to the observed tendency^26^ of midges to occasionally perform nearly harmonic oscillations conducted in synchrony with another midges. These pairwise interactions do not typically occur between midges that are nearest neighbours and are presumably mediated acoustically^26^. Being intrinsically linked to motor function, the flight tones should be reflected in the midge flight characteristics. This is evidenced in distributions of acceleration (Figs. 5, 6). The potential connection between acoustic ordering and the swarm structure warrants further investigation because if the modelling is to fully explain swarming, then it must be rooted in the correct biology, rather than in assumptions at the epi-phenomenological level. Embryo mathematical models for slab formation are presented in the Supplementary Material IV.

Intrinsic noise (i.e. thermal fluctuations) were shown to be crucial for the emergence of the thermodynamic properties of midge swarms, as prefigured by Reynolds^22^. This mirrors previous studies which have attributed the swarms’ emergent macroscopic mechanical properties, including for example, tensile strength and finite yield strength, to the presence of intrinsic multiplicative noise^17,18^. In this sense intrinsic noise, unlike internal slab structure, sculpts both the thermodynamic and mechanical properties of swarms. It does so by restructuring the basins of attraction (potential wells) that determine a swarm’s internal structure. Nonetheless, the thermodynamic, gas-like properties and the solid-like, mechanical properties emerge under different kinds of perturbations^4,27^, Sinhuber et al.^8,11,14^, i.e., the response of the swarms to external perturbations, whether gas-like or solid-like, is context dependent. These emergent properties are thus complementary rather than contradictory properties. In other words, external perturbations can drive phase transitions resulting in either gas-like or solid-like states. These emergent properties can be advantageous, functioning, for example, to suppress imposed perturbations very efficiently and keeping the swarm stable and stationary even in a noisy, stochastic environment^11^. Nonetheless, their emergence may be an accidental by-product of the swarm dynamics rather than the result of selection pressures for advantageous behaviours^11,16,17^. In the Supplementary Material VII & VIII I uncover two further accidental but potentially advantageous behaviours. I show that laboratory swarms of *Chironomus riparius* midges are close to being critically damped, i.e., are poised between being over- and underdamped. Consequently, these midges tend to return to their equilibrium position—the centre of the swarm—in the minimum time; typically, just failing to overshoot and not making single oscillations. With the aid of stochastic models, I show that critical damping in insect swarms does not require fine tuning, because it arises freely. I also report that insect swarms are predicted to behave like auxetic materials which have negative Poisson ratios, i.e., when swarms are pulled apart in one direction, as in Ni and Ouellette^4^, they expand rather than contract in the perpendicular direction. This suggests that swarms do not become inherently weaker when stretched because they thicken in response to a tensile force, rather than becoming thinner like positive-Poisson-ratio material.

Finally, the similitude with van der Waals gases suggests that insect swarms can undergo the equivalent of a liquid–gas phase transition (as prefigured in the Supplementary Material IV and complementing the analyses of Reynolds^16,22,28^ and van der Vaart et al.^11^) and that these two phases can coexist. [Insects in the bottom ‘compressed’ slab (Fig. 1) may correspond to liquid condensates that can form in van der Waals gases and which under the action gravity settle out.] This warrants further investigation, as does the potential application to other kinds of swarming insects [Supplementary Material IX].

## Supplementary Information

Supplementary Information.

## Data Availability

Computer codes can be obtained from the author.
