# Supplementary material for: Understanding the thermodynamic properties of insect swarms
Source: Sci Rep. 2021 Jul 22;11:14979. doi: 10.1038/s41598-021-94582-x (PMC8298516; doi:10.1038/s41598-021-94582-x)
Supplement: Supplementary file 1 — Supplementary Information. [file 41598_2021_94582_MOESM1_ESM.pdf]

Supplementary Material

I: No evidence for long-range interactions in laboratory insect swarms

Tetrahedra are the minimum configuration required to describe the 3-dimensional movements of midges (since 3 midges will always lie in a plane). Here I show that the statistical properties of these tetrahedra mirror expectations for Gaussian, independent individual positions; thereby bolstering the analysis of Puckett et al. [2014] who reported the acceleration measurements show a clear short-range repulsion but do not provide conclusive evidence of long-range interactions between individuals and their nearest neighbours.

Here the tetrahedra are studied using the approach of Pumir et al. [2000] and Biferale et al. [2005]. To characterize the shapes of the tetrahedra, it is useful to introduce the change of coordinates: $\boldsymbol{\rho}_0 = (\boldsymbol{x}_1 + \boldsymbol{x}_2 + \boldsymbol{x}_3 + \boldsymbol{x}_4)/2$, $\boldsymbol{\rho}_1 = (\boldsymbol{x}_2 - \boldsymbol{x}_1)/\sqrt{2}$, $\boldsymbol{\rho}_2 = (2\boldsymbol{x}_3 - \boldsymbol{x}_2 - \boldsymbol{x}_1)/\sqrt{6}$, $\boldsymbol{\rho}_3 = (3\boldsymbol{x}_4 - \boldsymbol{x}_3 - \boldsymbol{x}_2 - \boldsymbol{x}_1)/\sqrt{12}$ and to introduce the 'inertia-like' matrix $\boldsymbol{I} = \boldsymbol{\rho}^T\boldsymbol{\rho}$ where $\boldsymbol{\rho}$ is the square matrix $[\boldsymbol{\rho}_1, \boldsymbol{\rho}_2, \boldsymbol{\rho}_3]$ ($\boldsymbol{\rho}_1$, $\boldsymbol{\rho}_2$, $\boldsymbol{\rho}_3$ are a minimal representation of the shapes of tetrahedra; these shapes do not depend on the centre of mass, $\boldsymbol{\rho}_0$). The eigenvalues of $\boldsymbol{I}$, $g_1 \geq g_2 \geq g_3$ provide a way to characterise the shapes of the tetrahedra: $g_1 = g_2 = g_3$ corresponds to an isotropic object; $g_1 \approx g_2 \gg g_3$ corresponds to a pancakelike (coplanar) object; and $g_1 \gg g_2, g_3$ corresponds to a needle-like (co-linear) object. The tetrahedra within the midge swarms tend to have almost two-dimensional, strongly elongated geometries; exactly mirroring expectations for Gaussian, independent individual positions (Fig. S1). Moreover, these tetrahedra do not have a preferred spatial orientation. Distributions of the volumes, $V = \frac{1}{3}Det\rho = \frac{1}{3}\sqrt{g_1 g_2 g_3}$, of the tetrahedra also mirror expectations for Gaussian independent individual positions (Fig. S2). The tendency of midges and their nearest neighbours to form almost two-dimensional structures therefore has mostly an "entropic" origin.

Following Biferale et al. [2005] the dynamics of the tetrahedra can be analysed by introducing the relative velocities: $\boldsymbol{W}_1 = (\boldsymbol{u}_2 - \boldsymbol{u}_1)/\sqrt{2}$, $\boldsymbol{W}_2 = (2\boldsymbol{u}_3 - \boldsymbol{u}_2 - \boldsymbol{u}_1)/\sqrt{6}$, $W_3 = (3\boldsymbol{u}_4 - \boldsymbol{u}_3 - \boldsymbol{u}_2 - \boldsymbol{u}_1)/\sqrt{12}$. The evolution of a tetrahedron is given by

$$K \equiv \frac{1}{2}\frac{d}{dt}\rho\rho^T = \frac{1}{2}(W\rho^T + \rho W^T)$$

Geometrical information about these velocities can be obtained from the eigenvalues. $k_1 \geq k_2 \geq k_3$, of **K**. The velocities of the tetrahedra tend to have one extensional component and one compressional component: exactly mirroring expectations for Gaussian, independent individual positions (Fig. S3). Note also that the alignment of the eigenvectors associated with g_1 and k_1 mirrors expectations for Gaussian, independent individual positions; indicating that strong velocity differences are not preferentially associated with intense elongations approximately in the same direction.

Following Biferale et al. [2005] the dynamics of the tetrahedra were further examined by determining the average times, $\langle T(g_i) \rangle$ for the eigenvalues, g_1, g_2, g_3 to double (Figs.S4a and S5a). Midge swarms exhibit a scaling regime, $\langle T(g_i) \rangle \sim g_i^1$. The stochastic model of Reynolds et al. [2017] for the trajectories of non-interacting individuals, within which velocities and positions evolve jointly as Markovian process, predicts such a scaling regime, albeit with a scaling exponent of 2 rather than 1. The presence of the scaling regime and the self-similarity are more evident after applying simple multiplicative factors on the g-axis (Fig. S4b and Fig. S5b). The presence of a range where the doubling times for different eigenvalues are the same is equivalent to stating that the typical shape of the tetrahedra is preserved while its size increases. Stochastic modelling suggests that the difference between the observed and predicted scaling exponents cannot be attributed to short-range repulsion as reported by Puckett et al [2014]. Nor can it be attributed to interspecific variability of model parameters. These do not change the prediction for the scaling exponent. It may instead to be attributed to having presupposed that the acceleration autocorrelation approaches a δ function at the origin, corresponding to an uncorrelated (white noise) component in the acceleration, and hence a Markov process. When the white-noise in the model of Reynolds [2017] is replaced by noise with exponential autocorrelation, i.e., when the white-noise is replaced by an OU-process, the predicted scaling exponent can in brought into line with observations (Fig. S6). In such models, accelerations, velocities and positions are jointly Gaussian and evolve collectively as a Markov process [Reynolds and Ouellette 2016]. Predicted scaling exponents depend on model parameters, suggesting that the scaling is species dependent.

'Perceived velocity gradients' were examined following the approach reviewed in Pumir and Naso [2012 and references therein]. Perceived velocity gradients, M, are defined by minimizing the quantity $\Gamma = (W - \rho M)^2$. This gives $M = \rho^{-1} W$. M is characterized by two invariants, $R = -\frac{1}{3} Tr M^3$ and $Q = -\frac{1}{2} Tr M^2$. This is because the eigenvalues of M are given

by the roots of $\lambda^3 + Q\lambda + R = 0$. The quantity $\Delta = 4Q^3 + 27R^2$ separates purely real solutions ($\Delta < 0$), physically interpreted as the flow being locally non-rotational, from one real and a complex conjugate pair of roots ($\Delta > 0$), corresponding to a flow that is locally rotating The distribution of the two invariants, *R* and *Q*, mirror predictions from stochastic models for non-interacting individuals (Fig. S7). In accordance with observations, the model predicts that the flow is locally rotating 88% of the time. The $TrM \neq 0$ indicating that the flows are compressible. Observed and predicted values of TrM have heavy power-law tails; $P(TrM) \sim (TrM)^{-2}$.

The above approaches can in principle be extended to include accelerations, e.g. via $\frac{dK}{dt}$ and via perceived gradients in accelerations, $\rho^{-1}A$. It is, however, potentially problematic to compare observations with expectations for independent individuals derived from 1st-order stochastic trajectory models. This is simply because accelerations *per se*, as opposed to average accelerations, are not well-defined model quantities. Nonetheless, plots of k_1' against k_2' etc. (ala Fig. S3) where $k_1' \geq k_2' \geq k_3'$ are the eigenvalues of $\frac{dK}{dt}$ closely match model predictions.

References

Biferale, L., Boffetta, G., Celani, A., Devenish, B.J., Lanotte, A. & Toschi, F. Multiparticle dispersion in fully developed turbulence. *Phys. Fluids* **17**, 111701 (2005).

Puckett, J.G., Kelley, D.H. & Ouellette, N.T. Searching for effective forces in laboratory swarms. *Sci. Rep.* **4**, 4766 (2014).

Pumir, A. & Naso, A. Insight on turbulent flows from Lagrangian tetrads. *Comp. Ren. Phys.* **13***,* 889-989 (2012).

Pumir, A., Shraiman, B.I. & Chertkov, M. Geometry of Lagrangian Dispersion in Turbulence. *Phys. Rev. Lett*. **85**, 5324-5327 (2000).

Reynolds, A.M. & Ouellette, N.T. Swarm dynamics may give rise to Lévy flights. *Sci. Rep.* **6**, 30515 (2016).

Reynolds, A.M., Sinhuber, M. & Ouellette, N.T. Are midge swarms bound together by an effective velocity-dependent gravity? *Eur. Phys. J. E.* **40**, 46 (2017).

Sinhuber, M., van der Vaart, K., Ni, R., Puckett, J.G., Kelley, D.H. & Ouellette, N.T. Three-dimensional time-resolved trajectories from laboratory insect swarms. *Sci. Data* **6**, 190036 (2019).

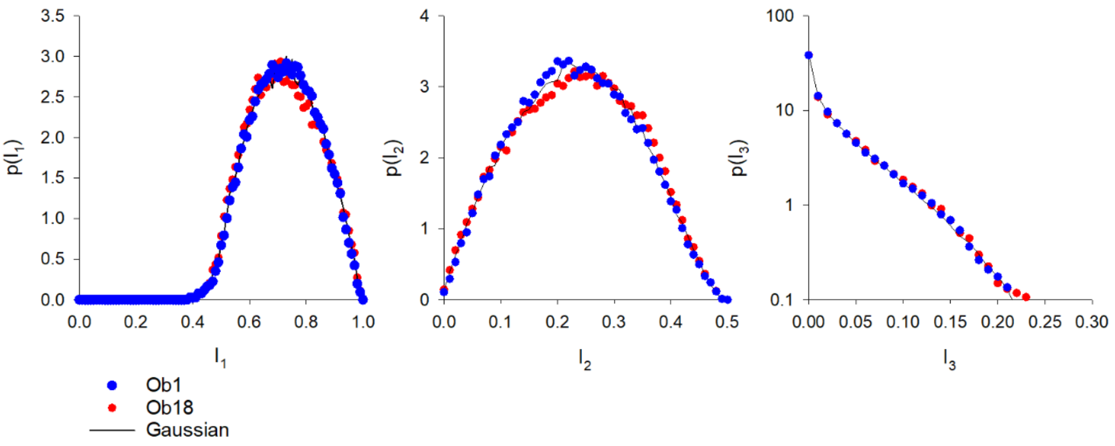

.

Figure S1. The tetrahedra within the midge swarms tend to have almost two-dimensional, strongly elongated geometries mirroring expectations for Gaussian, independent individual positions. Distributions of the dimensionless shape parameters $I_i = g_i/R^2$ where $\boldsymbol{R} = \sqrt{g_1 + g_2 + g_3}$ is the radius of gyration. Distributions are shown for swarms containing on average 94 (Ob1) and 19 (Ob18) individuals. Data are taken from Sinhuber et al. [2019]. Shown for comparison are simulation data for Gaussian, independent individual positions. Covariances $\langle g_i g_j \rangle$ also closely mirror expectations for Gaussian, independent individual positions.

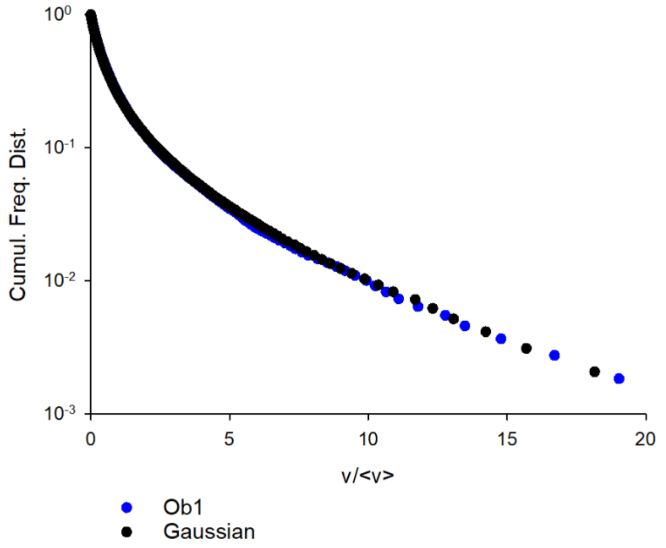

Figure S2. Distributions of space volumes have power-law tails that are indicative of self-similarity exactly mirroring expectations for Gaussian, independent individual positions. Data are shown for a swarm (Ob1) containing on average 94 individuals. Data are taken from Sinhuber et al. [2019].

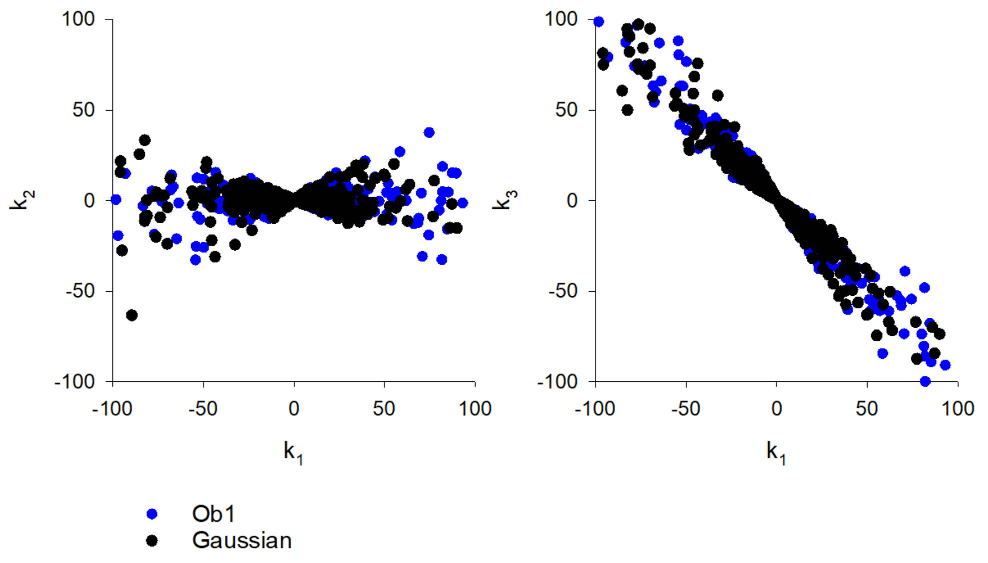

Figure S3. The velocities of the tetrahedra tend to have one extensional component and one compressional component exactly mirroring expectations for Gaussian, independent individual positions. Data are shown for a swarm (Ob1) containing on average 94 individuals. Data are taken from Sinhuber et al. [2019].

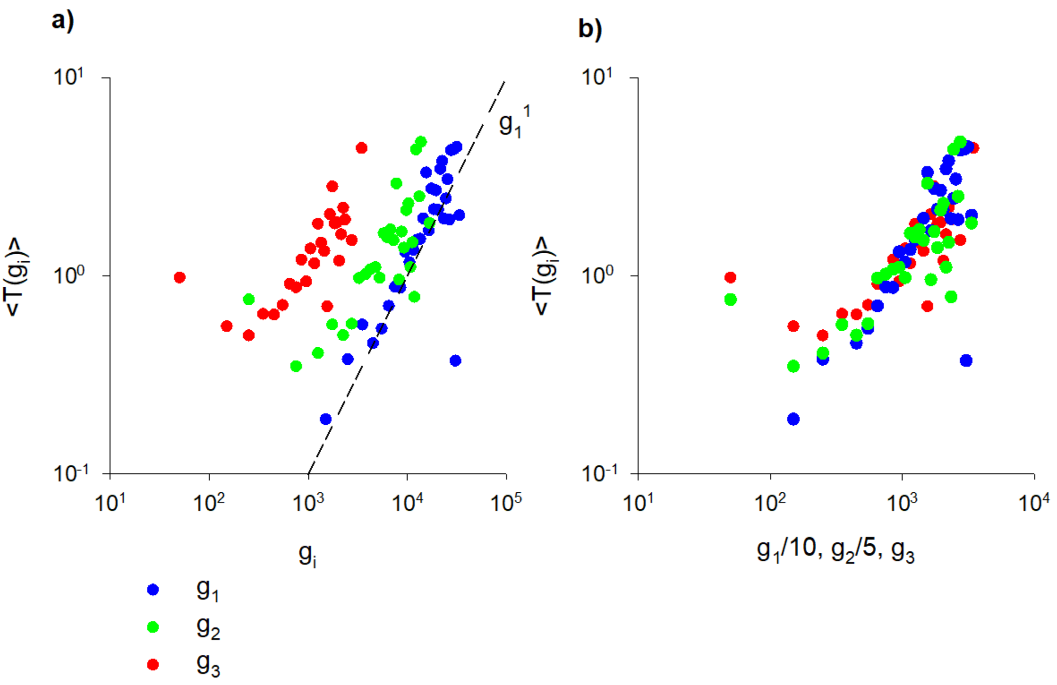

Figure S4. a) Doubling times for the eigenvalues, g_i, of the inertia matrix, $I = \rho^T \rho$. b) Same data rescaled on the horizontal axis with the proportions g_1:g_2:g_3=10:5:1. Data are shown for all 19 swarms in the dataset of Sinhuber et al. [2019].

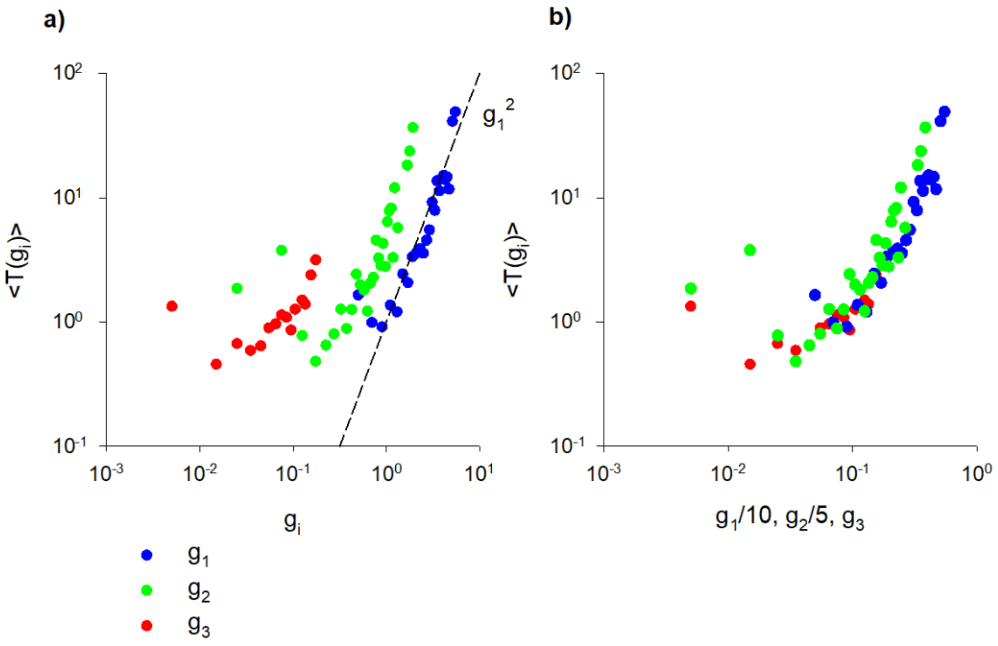

Figure S5. a) Predicted doubling times for the eigenvalues, g_i, of the inertia matrix, $I = \rho^T \rho$. b) Same data rescaled on the horizontal axis with the proportions g_1:g_2:g_3=10:5:1. The trajectories of N=100 non-interacting individuals with Gaussian positions and velocities were simulated simultaneously using the stochastic model of Reynolds et al. [2017] with $\sigma_R = 2, \sigma_u = 1, T = 1$. The same scaling relationship were obtained when model parameters including N were increased or decreased by a factor of 2. The same scaling exponent was obtained for individuals with Gaussian positions and exponential velocities.

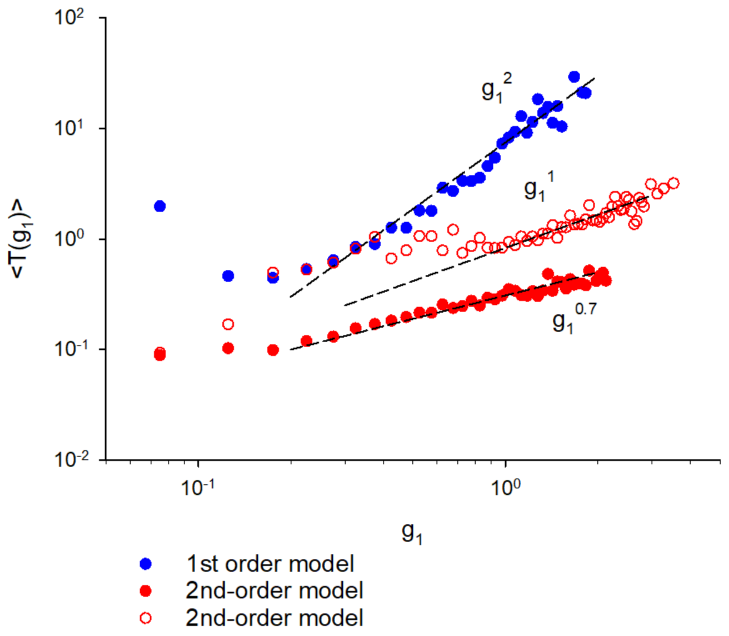

Figure S6. Predicted doubling times for the eigenvalues, g_i, of the inertia matrix, $\boldsymbol{I} = \boldsymbol{\rho}^T\boldsymbol{\rho}$. The trajectories of N=100 non-interacting individuals with Gaussian positions and velocities were simulated simultaneously using the stochastic model of Reynolds et al. [2017] with $\sigma_R = 1, \sigma_u = 1, T = 1$. Predictions are shown for the model driven by white noise and by exponentially correlated noise with autocorrelation timescale t_A=*0.1* (o) and 1.0.(•).

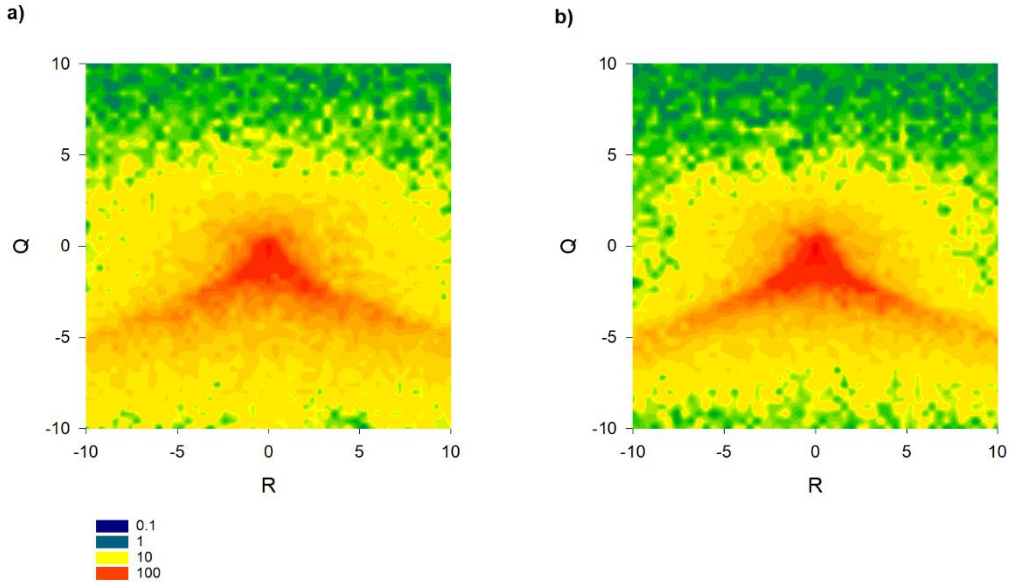

Figure S7. Observed (a) and predicted (b) distributions of the two invariants of the perceived velocity gradients, $R = -\frac{1}{3}TrM^3$ and $Q = -\frac{1}{2}TrM^2$. Data are pooled from all 19 swarms in the dataset of Sinhuber et al. [2019]. Predictions are shown for a swarm containing 100 non-interacting individuals. Predictions were obtained using the stochastic model of Reynolds et al. [2017] with all parameters set to unity.

II: Indirect speculative evidence of confinement to horizontal slabs

Slab confinement can be assessed quantitively following the methodology of Pumir et al. [2000] and Biferal et al. [2005] since 4 points along an individual's trajectory will form a tetrahedron. At short times, individual trajectories are, as expected, colinear (as $g_1 \gg g_2, g_3$) but at longer times they are coplanar (as $g_1 \approx g_2 \gg g_3$); and as expected mirror expectations for Gaussian, independent positions (Figs. S8). The tendency to form almost two-dimensional structures therefore has mostly an "entropic" origin: indeed, there are many pancake-like tetrahedra (very small I_3) when positions are decorrelated. At long times the proportions of midge trajectories elongated in the x-, y- and z-directions are approximately 5:5:1, indicating that confinement is mostly on horizontal planes.

In this regard, it is interesting to note that Cavagna et al. [2017] showed that wild swarms appear to belong to a novel dynamical universality class: as correlations exhibit scaling behaviour that is atypical 3-dimensional systems. The observed scaling is distinctly different from the scaling that characterizes the 3-dimensional Vicsek et al. [1995] model of swarming self-propelled particles and much more like the scaling that characterizes the 2-dimensional form of this model [Baglietto and Albano 2008]. That is, the apparent novelty of the scaling may be attributed to slab confinement.

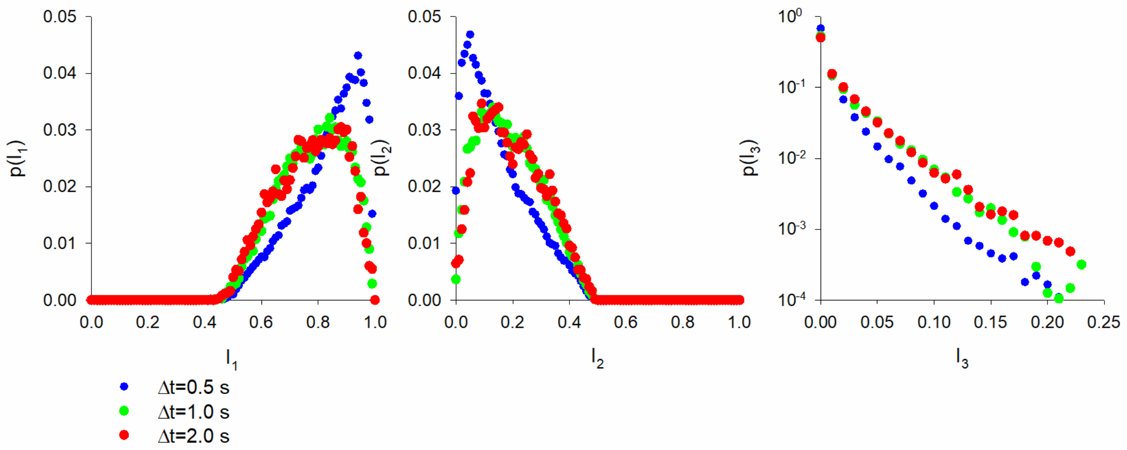

Figure S8. Individual trajectories are colinear at short times and coplanar at long times. Distributions of the dimensionless shape parameters $I_i = g_i/R^2$ where $\boldsymbol{R} = \sqrt{g_1 + g_2 + g_3}$ is the radius of gyration. Data are taken from Sinhuber et al. [2019]: swarm Ob1 which on average contains 94 individuals.

References

Baglietto, G. & Albano, E.V. Finite-size scaling analysis and dynamic study of the critical behavior of a model for the collective displacement of self-driven individuals. *Phys. Rev. E* **78**, 021125 (2008).

Biferale, L., Boffetta, G., Celani, A., Devenish, B.J., Lanotte, A. & Toschi, F. Multiparticle dispersion in fully developed turbulence. *Phys. Fluids* **17**, 111701 (2005).

Cavagna, A. et al. Dynamic scaling in wild swarms. *Nat. Phys.* **13**, 914-918 (2017).

Pumir, A., Shraiman, B.I. & Chertkov, M. Geometry of Lagrangian Dispersion in Turbulence. *Phys. Rev. Lett*. **85**, 5324-5327 (2000).

Vicsek, T., Czirók, A., Ben-Jacob, E., Cohen, I. & Shochet, O. Novel type of phase transition in a system of self-driven particles. *Phys. Rev. Lett.* **75**, 1226-1229 (1995).

III: Slab structure does not develop systematically with swarm size for arbitrarily defined density profiles - associated mean accelerations are non-physical

The density profiles of laboratory swarms are skewed perhaps because of the energetic demands of flying upwards and/or because they are bounded at ground level [Kelley and Ouellette 2013]. Such density profiles could for argument sake be crudely represented as mono-modal, gamma distributions $p(z) = \frac{1}{\Gamma(k)\theta^k} z^{k-1} e^{-z/\theta}$. Here I show that the number of Gaussians in the best representations of an such arbitrarily defined density profile does not increase monotonically with swarm size, contrary to the analysis of natural swarms (Fig. 3). I also show that the mean accelerations associated with such mono-modal distributions are non-physical and incompatible with observations. These results are consistent with the claims in the main text that the multi-Gaussian fits to the swarm density profiles are indicative of actual structure and with swarm growth proceeding slab by slab.

As for natural swarms (see main text) the best representation of the putative gamma distributions in terms of multi-Gaussians was found using maximum likelihood method and the best multi-Gaussian distribution was identified objectively using the Akaike information criterion. Typically, the number of Gaussians in the best representation does not increase monotonically with swarm size (Fig. S9).

The mean acceleration (effective force) of an individual in the vertical direction and the density profile $p(z)$ are related by $\langle A_z|z\rangle = -\int_{-\infty}^{w} \hat{p}(w)dw \frac{d}{dz} lnp(z)$ where $\hat{p}(w)$ is the distribution of vertical velocities [Reynolds and Ouellette 2016]. For gamma distributions $\langle A_z|z\rangle = -\int_{-\infty}^{w} \hat{p}(w)dw \left(\frac{k-1}{z} - \frac{1}{\theta}\right)$. The singularity at z=0 is non-physical and incompatible with the observed near linear form [Okubo 1986, Kelley and Ouellette 2013]; a form predicted by the multi-Gaussian representation of the density profile (Fig. S10). Singularities arise in other putative skewed density profiles such as beta prime, chi-squared and Rayleigh distributions.

References

Kelley, D.H. & Ouellette, N.T. Emergent dynamics of laboratory insect swarms. *Sci. Rep.* **3**, 1073, 1-7 (2013).

Okubo, A. Dynamical aspects of animal grouping: swarms, schools, flocks, and herds. *Adv. Biophys*. **22**, 1-94 (1986).

Reynolds, A.M. & Ouellette, N.T. Swarm dynamics may give rise to Lévy flights. *Sci. Rep.* **6**, 30515 (2016).

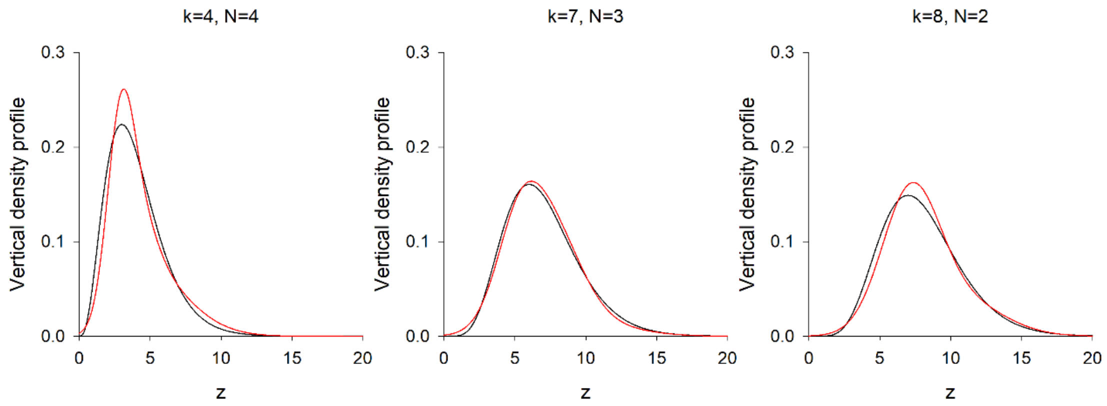

Figure S9. Slab structure does not develop systematically with swarm size for arbitrarily defined density profiles. Results are shown for gamma distributions $p(z) = \frac{1}{\Gamma(k)\theta^k} z^{k-1} e^{-z/\theta}$ with *θ=1* (black lines). The number of Gaussians, *N*, in the best fit multi-Gaussian representations (red lines) does not increase monotonically with increasing width (k) of the distribution.

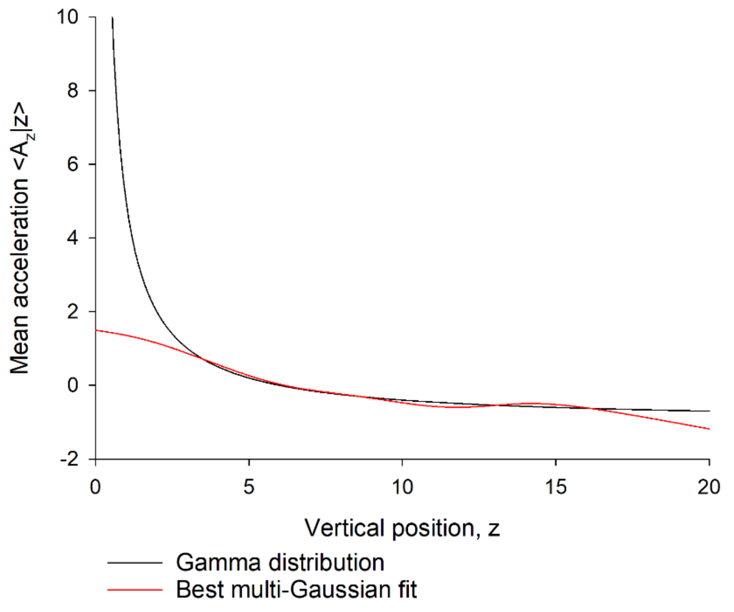

Figure S10. Mean accelerations associated with arbitrarily defined density profiles unlike those associated with their multi-Gaussian representation do not resemble the observed near-linear forms. Results are shown for a gamma distribution $p(z) = \frac{1}{\Gamma(k)\theta^k} z^{k-1} e^{-z/\theta}$ with *k*=*3* and *θ*=*1* (black line) and for the best fit tri-Gaussian (see Fig. S9). Velocities are taken to be Gaussian distributed with mean zero and unit variance.

IV: Speculative ideas about slab formation

Model 1

In this model, as the population size of the swarm increases, existing slabs move apart to accommodate more individuals and in doing so become less stable (become noisier). This noise creates new centres of attraction, spawning 'embryonic' slabs and is thereby constructive. The swarm population then redistributes, re-establishing equilibrium, and the intrinsic noise diminishes. The cycle then repeats. In this regard it is interesting to note that swarms are analogous to self-gravitating systems [Okubo 1986, Reynolds 2019a and references therein] and that the model of slab formation is analogous to Hoyle's Steady State Model. Near constancy of average density is a hallmark of laboratory swarms [Kelley and Ouellette 2014]. Motivation for the approach comes from: the association of tensile strength with intrinsic noise [Reynolds 2019b]; the subsequent identification of intrinsic noise in quiescent swarms [Reynolds 2021]; the presence of an embryonic-like slab located about mid-way between two more prominent slabs in laboratory data (Fig. 1b main text).

A swarm with equi-spaced, equi-size slabs can be represented in simple stochastic models by a periodic potential

$$U(z) = k_0 sin(z) \quad \text{(S1)}$$

since the associated vertical density profile is

$$p(z) = \exp\left(-k_0 sin(z)\right) \quad \text{(S2)}$$

[Reynolds and Ouellette 2016, Supplementary Material V]. The potential wells can be expected to be noisy. For simplicity and for illustrative purposes it is instructive to consider the case of fast, spatially-independent stochastic variability so that the strength of the potential because $k = k_0 + k_1(t)$ where $k_1(t)$ is a white noise component such that $\langle k_1(t)k_1(t^{/})\rangle = 2F\delta_{t,t^{/}}$. At long times (compared with the velocity autocorrelation timescale) vertical movements can be modelled stochastically by

$$dz = -\cos(z)\left(k_0 dt + \sqrt{2F}d\xi_1\right) + \sqrt{2}d\xi_2 \quad \text{(S3)}$$

where $d\xi_1$ and $d\xi_2$ are independent white noise processes. [For details of model formulation see Reynolds and Ouellette 2016, Reynolds 2019b, Supplementary Material V]. As expected, the noise changes the shape of the potential well [Reynolds 2019b], resulting in new minima that lie between the original minima (Fig. S11). The equilibrium vertical density profile now becomes

$$p(z) = \left(\sqrt{F(F+1)} - Fsin(z)\right)^{\frac{1}{2\sqrt{F(F+1)}}-1} \left(\sqrt{F(F+1)} + Fsin(z)\right)^{-\frac{1}{2\sqrt{F(F+1)}}-1} \quad \text{(S4)}$$

The emergent ‘embryonic’ slabs could seed new centres of attraction that are directly analogous to the original centres.

Attanasi et al. [2014] posited that wild swarms are at near criticality, i.e., poised at the cusp of a phase transition. This was motivated by the detection of scaling behaviour in correlation functions; behaviour that was subsequently identified in simulation data for swarms of non-interacting interactions [van der Vaart et al. 2020]. Although the original motivation is unjustified (and although putative phases were not identified), the above analysis again raises the spectre of near criticality because the number of slabs is predicted to be near to a doubling transition; more generally near a slab-creation transition.

Model 2

In this model, increases in acoustic interference that accompany increases in population size are offset by increases acoustic clustering [Aldersley et al. 2017] and so by increases in the number of slabs. As the number of slab increases, intrinsic noise naturally causes the physical size of the swarm to increase thereby maintaining near-constancy of the average density. In the analogy with self-gravitating systems, this is analogous to Hoyle’s Steady State Model. Near constancy of average density is a hallmark of laboratory swarms [Kelley and Ouellette 2014]. The model predicts, as observed, that small (laboratory) swarms are weakly axisymmetric [Kelley and Ouellette 2014] whereas large (wild) swarms are cylindrical [Attanasi et al. 2014]. In accordance with observations [Sinhuber, Private Communication] this model predicts that external noise can cause explosive growth of the swarm.

Compared with model 1, this model is more realistic because simulated density profiles are spatially localized. It does not appear to be analytically tractable when F>0.

Vertical movements are modelled stochastically by

$$dz = \Omega \sin(\Omega z)\left(dt + \sqrt{2F} d\xi_1\right) - z dt + \sqrt{2} d\xi_2 \quad \text{(S5)}$$

[For details of model formulation see Reynolds and Ouellette 2016, Reynolds 2019b, Supplementary Material V]. For *F=0*, the equilibrium density profile is composed on equi-spaced, equi-size slabs that lie within a swarm having a Gaussian envelope,

$$p(z) = \exp\left(-cos(z\Omega) - \frac{z^2}{2}\right) \qquad \text{(S6)}$$

(Fig. S12). Intrinsic noise, which here acts on the slabs rather than on the confining potential, causes the swarm size to grow exponentially with the number of slabs (Figs. S13); thereby providing a mechanism for the regularization of the average density. By inducing acoustic clustering, acoustic interference can thus cause explosive growth of the swarm.

Adaptation

Finally, it is worth remarking that 'adaptation' may also be implicated in slab formation. As noted by Gorbonos et al. [2016] midges interact acoustically and the perception of sound may not be fixed but instead adapts to the local sound intensity so that acoustic intensity drops when there is a strong background noise; preventing damage and saturation of the sensory organs. Gorbonos et al. [2016] utilized the similarity in form between the decay of acoustic and gravitational sources to construct an 'adaptive gravity' model of swarming that agrees well with experimental observations of laboratory swarms. In the outskirts of a swarm, where the background noise is weak, the effects of adaptivity on the cohesive forces that bind the swarm together are weak. But in the centres of large swarms where the background noise is strong, adaptivity can sufficiently weaken the cohesive force that holds the swarm together. This led Gorbonos et al. [2016] to speculate on the appearance of a maximal swarm size, beyond which they become unstable and split. Here, this possibility is examined within the context of stochastic models of the trajectories of swarming insects.

Midges are, to good approximation, bound to the centre of the swarm by an effective mean acceleration (mean force) that grows linearly with distance from the swarm centre [Okubo 1986, Kelley and Ouellette 2013]. This is consistent with the results of stochastic model which predicts that the effective mean acceleration is given by $A = -\frac{\sigma_u^2}{\sigma_x^2}x$ when, as observed, the swarm density profile, $p(x)$, and velocities are Gaussian distributed, where *x* is the distance from the swarm centre [Reynolds and Ouellette 2016]. More generally, $A = \sigma_u^2 \frac{dlnp}{dx}$. Adaptation is expected to reduce effective mean accelerations within the cores of swarms ($|x| < \Lambda$) so, for example,

$$A = -\frac{\sigma_u^2}{\sigma_x^2}\frac{x^3}{\Lambda^2+x^2} \tag{S7}$$

In this case the equilibrium density profiles is given by

$$p(x) = Nexp\left(-\frac{x^2}{2\sigma_x^2}\right)\left(1+\frac{x^2}{\Lambda^2}\right)^{\frac{\Lambda^2}{2\sigma_x^2}} \tag{S8}$$

where *N* is a normalization factor. Adaptation thereby results in a near flat density profile within the core of the swarm (Fig. S14a). This increases the likelihood that the swarm will split because fluctuations in number density are now more likely to result in the spontaneous appearance of clusters of individuals away from the centre of the swarm and so result in the spontaneous appearance of new centres of attraction (Fig. S14b).

Note also that according to Eqn. S8, the physical size of the swarm increases as the strength of the adaption increases, i.e., increases as the population size and so the background noise increases. This is consistent with Gorbonos and Gov [2017] who reported that adaption stabilizes swarms against 'gravitational' collapse (Jean's instability). And it is consistent with the observed near constancy of the number density of *Chironomus riparius* midges in laboratory swarms [Kelley and Ouellette 2013].

References

Aldersley, A. et al. Emergent acoustic order in arrays of mosquitoes. *Curr. Biol.* **27**, R1193-R1213 (2017).

Attanasi, A. et al. Collective Behaviour without collective order in wild swarms of midges. *PLoS Comp. Biol.* **10**, e1003697 (2014).

Gorbonos, et al. Long-range acoustic interactions in insect swarms: an adaptive gravity model. *New. J. Phys.* **18**, 073042 (2016).

Gorbonos, D. & Gov, N.S. Stable Swarming Using Adaptive Long-range Interactions. *Phys. Rev. E* **95**, 042405 (2017).

Hoyle, F. A New Model for the Expanding Universe, *Mon. Not. Roy. Ast. Soc.* **108** 372-382. (1948).

Kelley, D.H. & Ouellette, N.T. Emergent dynamics of laboratory insect swarms. *Sci. Rep.* **3**, 1073, 1-7 (2013).

Okubo, A. Dynamical aspects of animal grouping: swarms, schools, flocks, and herds. *Adv. Biophys*. **22**, 1-94 (1986).

Reynolds, A.M. & Ouellette, N.T. Swarm dynamics may give rise to Lévy flights. *Sci. Rep.* **6**, 30515 (2016).

Reynolds, A.M. On the emergence of gravitational-like forces in insect swarms. *J. Roy. Soc. Int.* **16,** 20190404 (2019a).

Reynolds, A.M. On the origin of the tensile strength of insect swarms. *Phys. Biol.* **16** 046002 (2019b).

Reynolds, A.M. Intrinsic stochasticity and the emergence of collective behaviours in insect swarms. *Eur. Phys. J. E* **44,** 22 (2021).

van der Vaart, K., Sinhuber, M., Reynolds, A.M. & Ouellette, N.T. Environmental perturbations induce correlations in midge swarms. *J. Roy. Soc. Int.* **17**, 20200018 (2020).

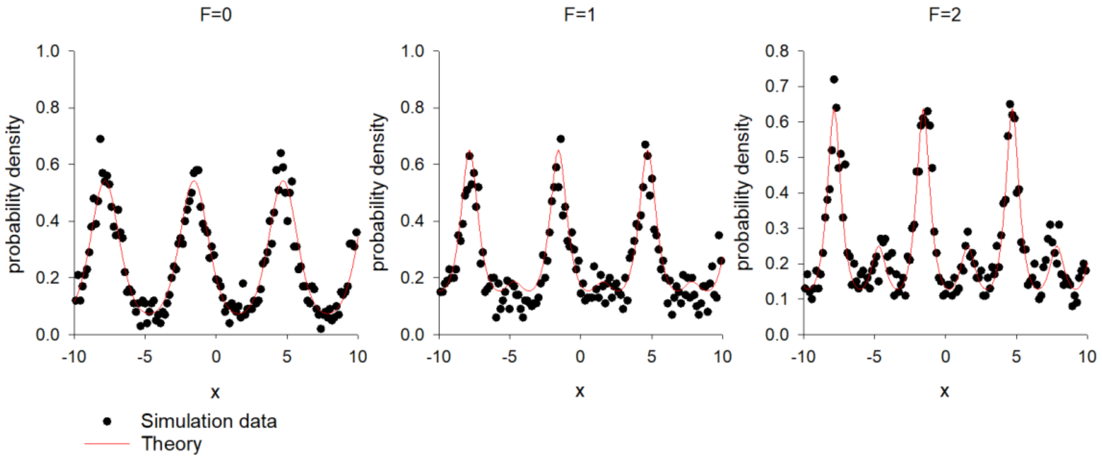

Figure S11. Intrinsic noise is constructive, seeding embryonic slabs. New slabs are seen to form between established slabs as the noise (F) increases. Simulation data was obtained with the random walk mode, Eqn. S3, with k_0=1 is shown together with the analytical predictions, Eqn. S4.

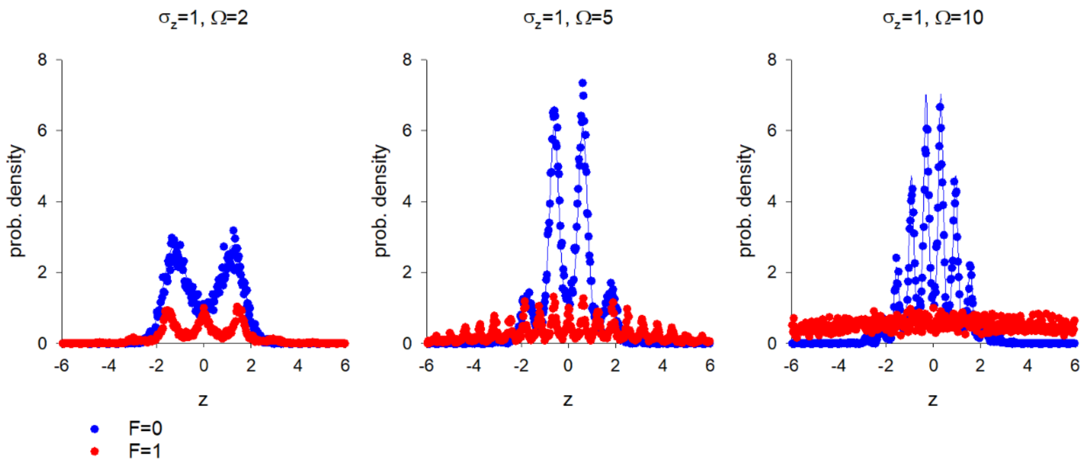

Figure S12. Equilibrium density profiles produced by the stochastic model, Eqn. S5, in the absence (•) and in the presence of intrinsic noise (•). Analytic predictions, Eqn. S6, (solid line) are shown for comparison. Intrinsic noise causes the swarm size to increase as the number of slabs increases.

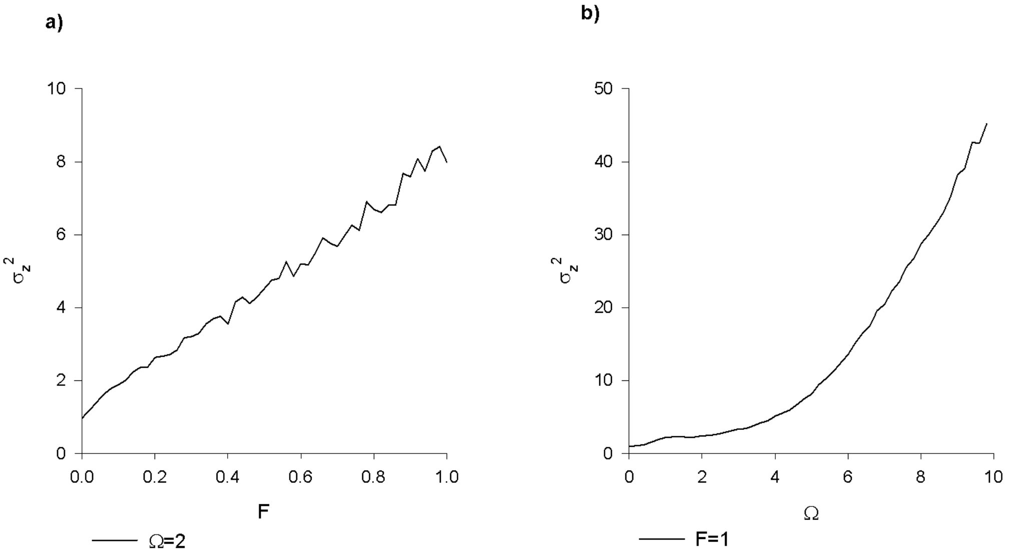

Figure S13. Predicted growth of swarm size with (a) increasing intrinsic noise and (b) with increasing number of slabs (Ω is the density of slabs).

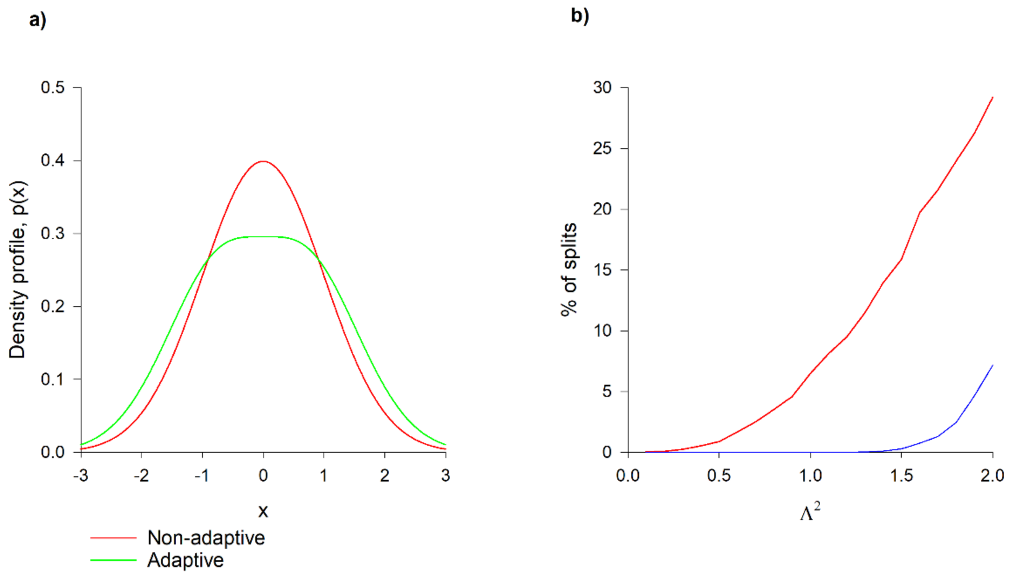

Figure S14a). Adaptation is predicted to flatten density profiles within the cores of swarms. b). Adaptation increases the potential for splitting. The likelihood that there are more individuals located outside the core, $|x| > 1$ than within it as a function of Λ^2. Results are shown for a swarm of size $\sigma_x = 1$ containing 100 (red line) and 1000 (blue line) individuals. Positions were drawn at random from the distribution, Eqn. S8.

V: Outline of stochastic model formulation

The formation of stochastic models for the trajectories of swarming insects was originally presented in Reynolds and Ouellette [2016] and is, here, outlined for completeness. The simplest such models, like Eqn. 4 in the main text, assume that the positions, z, of individual insects can be described by the stochastic differential equation

$$dz = a(z,t)dt + b(z,t)d\xi(t) \tag{S9}$$

where $d\xi(t)$ is an incremental Wiener process with correlation property $\overline{d\xi(t)d\xi(t+\tau)} = \delta(\tau)dt$. The deterministic term, $a(z,t)$, is determined by the requirement that the statistical properties of the simulated positions be consistent with a prescribed density profile, $p(x)$. Mathematically, this consistency condition, under the Ito interpretation of the noise term, requires that $a(x,t)$ be a solution of the Fokker-Planck equation

$$\boldsymbol{\frac{\partial P}{\partial t}} = -\boldsymbol{\frac{\partial}{\partial z}}(\boldsymbol{a}p) + \boldsymbol{\frac{1}{2}\frac{\partial^2}{\partial z^2}}(b^2 p) \tag{S10}$$

Therefore,

$$ap = \frac{1}{2}\frac{\partial}{\partial z}(b^2 p) \tag{S11}$$

for statistically stationary swarms having, $\frac{\partial p}{\partial t} = 0$.

It follows that for swarms with Gaussian density profiles, i.e. with $p = \frac{1}{\sqrt{2\pi}\sigma_z} exp\left(-\frac{(z-\bar{z})^2}{\sigma_z^2}\right)$, and having purely additive (position-independent noise), $b = \sqrt{2}F$ where F is a constant, that

$$dz = -F^2\left(\frac{z-\bar{z}}{\sigma_z^2}\right)dt + \sqrt{2}FdW \tag{S12}$$

Stochastic models for the joint evolution of an individuals' position and velocity are formulated in an exactly analogous way [Reynolds and Ouellette 2016].

References

Reynolds, A.M. & Ouellette, N.T. Swarm dynamics may give rise to Lévy flights. *Sci. Rep.* **6**, 30515 (2016).

VI: Interpreting the pressure-density relation for the 'vapour phase'

Laboratory insect swarms consist of a core 'condensed' phase surrounded by a dilute 'vapour' phase [Sinhuber and Ouellette 2017]. These two phases coexist in equilibrium but have distinct macroscopic properties. In the condensed phase, pressure, *P*, is approximately proportional to density, *n*, (i.e., its 'isothermal') whereas in the vapour phase $P \propto n^{\xi}$ with $\xi \approx 1/2$ [as predicted by the model of Reynolds et al. 2018a]. The vapour phase relationship does not have a thermodynamic analogue, but it does have a gravitational analogue, corresponding to descriptions of interstellar dust clouds [Shu et al. 1972, Viala 1972], as prefigured by Reynolds [2021]. The core is analogous to the globular cluster [Reynolds 2018b], as anticipated by Gorbonos et al. [2016]. The association of the vapour phase with interstellar dust clouds adds to a long-standing [Okubo 1986] and productive analogy between insect swarms and self-gravitating systems [Reynolds 2018b, 2019, 2020, Gorbonos et al. 2016, Gorbonos and Gov 2017, Gorbonos et al. 2020]. In this regard it is interesting to note the smallest swarms (with less than 10 individuals) are predicted to lack condense cores and be entirely analogous to interstellar dust clouds [Reynolds 2021].

References

Gorbonos, D., Ianconescu, R., Puckett, J.G., Ni, R., Ouellette, N.T. & Gov, N.S. Long-range acoustic interactions in insect swarms: an adaptive gravity model. *New J. Phys.* **18**, article 073042 (2016).

Gorbonos, D. & Gov, N.S. Stable Swarming Using Adaptive Long-range Interactions. *Phys. Rev. E* **95**, 042405 (2017).

Gorbonos, D., van der Vaart, K., Sinhuber, M., Puckett, J.G., Reynolds, A.M., Ouellette, N.T. & Gov. N.S. Similarities between insect swarms and isothermal globular clusters. *Phy. Rev. Res.* **2**, 013271 (2020).

Okubo, A. Dynamical aspects of animal grouping: swarms, schools, flocks and herds. *Adv. Biophys.* **22**, 1-94 (1986).

Reynolds, A.M. Langevin dynamics encapsulate the microscopic and emergent macroscopic properties of midge swarms. *J. Roy. Soc. Inter.* **15**, 20170806 (2018a).

Reynolds, A.M. Fluctuating environments drive insect swarms into a new state that is robust to perturbations. *Europhys. Lett.* **124**, 38001 (2018b).

Reynolds, A.M. On the emergence of gravitational-like forces in insect swarms. *J. Roy. Soc. Int.* **16,** 20190404 (2019).

Reynolds, A.M. Insect swarms can be bound together by repulsive forces. *Euro. Phys. J. E.* **43**, 39 (2020).

Reynolds, A.M. Intrinsic stochasticity and the emergence of collective behaviours in insect swarms. *Euro. Phys. J. E.* **44**, 22 (2021).

Shu, F.H., Milione, V., Gebel, W., Yuan, C., Goldsmith, D.W. & Roberts, W.W. Galactic shocks in an interstellar medium with two stable phases. *Astrophys. J.* **173**, 557-592 (1972).

Sinhuber, M. & Ouellette, N.T. Phase coexistence in insect swarms. *Phys. Rev. Lett.* **119**, 178003 (2017).

Viala, Y. Structure et critères d'effondrement gravitationnel d'une sphère polytropique d'indice négatif. *Comp. Rend. Acad. Sci. (Paris)* **275B**, 117-120, (1972).

VII: Critical Damping

Here I show that laboratory swarms of *Chironomus riparius* midges are critically damped and I show that critical damping arises freely in simple 3-dimensional stochastic models.

Laboratory swarms of *Chironomus riparius* midges [Sinhuber et al. 2019] are found to be either weakly overdamped (as the negative lobe is barely evident) or weakly underdamped (as there is no evidence of a secondary positive lobe on the velocity autocorrelation functions, i.e., the midges just fail to overshoot) (Fig. S15). Moreover, estimates for the frequencies of oscillatory modes are typically close to zero. This suggests that the laboratory swarms are close to being critically damped, poised between being over- and underdamped; contrary to the observations of laboratory swarms of *Anarete* midges [Okubo 1986] and observations of wild swarms of *Anopheles gambiae* [Butail et al. 2013]. Consequently, the *Chironomus riparius* midges tend to return to their equilibrium position – the centre of the swarm – in the minimum time; typically, just failing to overshoot and not making single oscillations. A precursor of this critical damping can be found in Kelley and Ouellette [2013] who showed that mean-squared displacements saturates when $\langle d^2 \rangle \approx R_s^2$; i.e., when the average midge has reached the edge of the swarm.

Critical damping seemingly requires fine tuning. It occurs in 1-dimensional models for swarms with Gaussian position and velocity statistics when $2\sigma_u^2 T = \sigma_x^2$ [Okubo 1986]. 3-dimensional models do not appear to be not analytically tractable. Nonetheless, the results of numerical simulations indicate that overshooting (resulting in a secondary positive lobe in the velocity autocorrelation) is generally absent or weak in the 3-dimensional versions of Okubo's [1986] model [Reynolds et al. 2017] when 'spin' (a preferred sense of rotation of individual trajectories) is the absent or is sufficiently weak (Fig. S16). [This is also true of second-order models wherein accelerations, velocities and positions rather than just velocities and positions evolve jointly as Markovian processes. And it is true of simulated spinless swarms as they relax back to their equilibrium positions after being displaced]. Stochastic models with spin produce looping trajectories [Borgas et al. 1997]. Spin has been detected in laboratory swarms of *Chironomus riparius* midges [Reynolds 2019, see also Reynolds 2020] but it may be stronger in laboratory swarms of *Anarete* midges [Okubo 1986] and in wild swarms of *Anopheles gambiae* [Butail et al. 2013]. Spin suppresses dispersion, thereby promoting cohesiveness but impedes a swarms' return to its' equilibrium form after being perturbed. This may account for the observations of Butail et al. [2013] who reported that the frequencies of oscillation increased with mean wind speed: environmental disturbances may excite the spin degrees of freedom because spin pushes the swarm into a more robust state. Robustness

may be further enhanced by the formation of transient, local order (synchronized subgroups) [Butail et al. 2013, Shishika et al. 2014, Reynolds 2019].

Critical damping (or nearly so) also arises freely in 2-dimensional stochastic models and is evident at the population level in two species of zooplankton, *Daphnia* and *Temora*, that swarm around light shafts [Banas et al. 2003]. Individual velocities decorrelate almost entirely in less than one period of the harmonic attractive force.

References

Banas, N.S. Wang D-P. & J. Yen. Experimental validation of an individual-based model for zooplankton swarming. In Handbook of Scaling Methods in Aquatic Ecology: Measurement, Analysis, Simulation. Edited by P.G. Strutton and L. Seuront. CRC Press pp161-180 (2003).

Borgas, M. Flesch, T.K., Sawford, B.L. Turbulent dispersion with broken reflectional symmetry. *Bound-Layer Meteor.* **332***,* 141-156 (1997).

Butail, S., Manoukis, N.C., Diallo, M., Ribeiro, J.M.C & Paley, D.A. The dance of male *Anopheles gambiae* in wild mating swarms. *J. Med. Ent.* **50**, 552-559 (2013).

Kelley, D.H. & Ouellette, N.T. Emergent dynamics of laboratory insect swarms. *Sci. Rep.* **3**, 1073, 1-7 (2013).

Okubo, A. Dynamical aspects of animal grouping: swarms, schools, flocks, and herds. *Adv. Biophys*. **22**, 1-94 (1986).

Reynolds, A.M. Fluctuating environments drive insect swarms into a new state that is robust to perturbations. *Europhys. Lett.* **124**, 38001 (2018).

Reynolds, A.M. On the emergence of gravitational-like forces in insect swarms. *J. Roy. Soc. Inter.* **16,** 20190404 (2019).

Reynolds, A.M. Insect swarms can be bound together by repulsive forces. *Eur. Phy. J. E.* **43**, 30 (2020).

Shishika, D., Manoukis, N.C., Butail, S. & Paley, D.A. Male motion coordination in anopheline mating systems. *Sci. Rep.* **4**, 6318 (2014).

Sinhuber, M., van der Vaart, K., Ni, R., Puckett, J.G., Kelley, D.H. & Ouellette, N.T. Three-dimensional time-resolved trajectories from laboratory insect swarms. *Sci. Data* **6**, 190036 (2019).

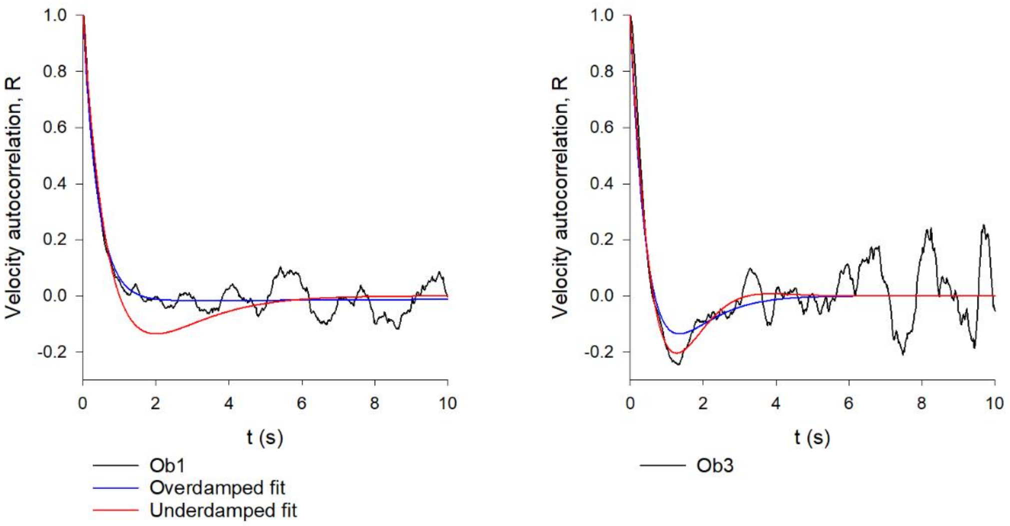

Figure S15. Examples of over- and underdamped swarms together with best fit velocity autocorrelations for Okubo's [1986] 1-dimensional model. Data are taken from Sinhuber et al. [2019].

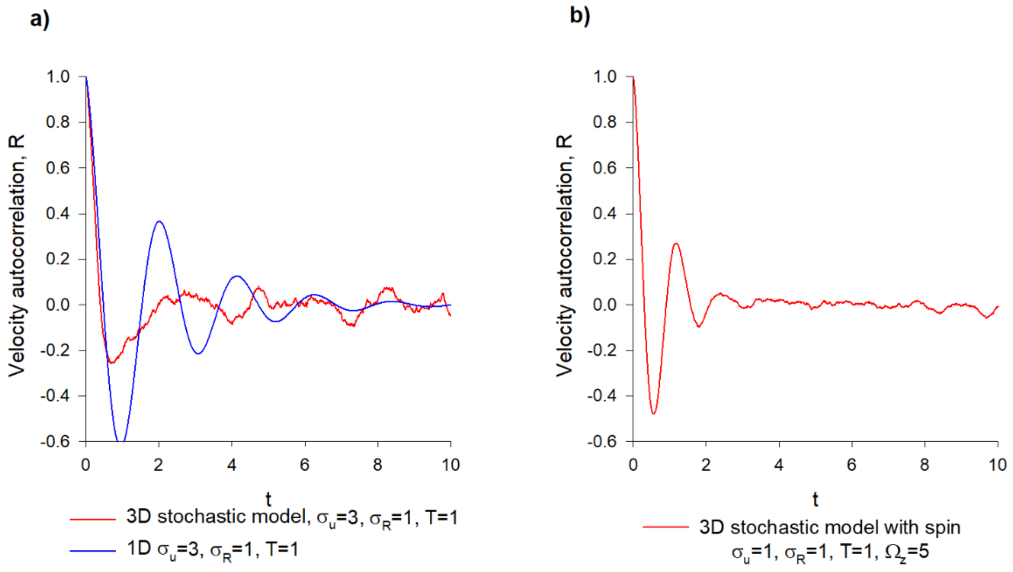

Figure S16. Near critical dampening arises freely in 3-dimensional stochastic models when spin is sufficiently weak [Okubo 1986, Reynolds et al. 2017, Reynolds 2019]. It occurs for many values of the model parameters.

VIII: Anticipated emergence of auxetic material-like properties

Laboratory swarms of the midge *Chironomus riparius* have macroscopic mechanical properties similar to solids, including a finite Young's modulus and yield strength [Ni and Ouellette 2016]. Reynolds [2019] showed somewhat counterintuitively that the emergence of these properties can be attributed to the presence of intrinsic noise. Here I remark that the analysis of Reynolds [2019] is readily extended from 1- to 2-dimensions and that 2-dimensional analysis predicts that the swarms behave like auxetic materials which have negative Poisson ratios, i.e., when swarms are pulled apart in one direction, as in Ni and Ouellette [2016], they expand rather than contract in the perpendicular direction. This suggests that swarms do not become inherently weaker when stretched because they thicken in response to a tensile force, rather than becoming thinner like positive-Poisson-ratio material.

Auxetic material-like properties are predicted to arise because stretching a swarm in one direction will, by virtue of diluting the population, tend to increase the fluctuations in the resultant centrally attractive force. Such fluctuations (intrinsic noise) will tend to further increase the overall size of swarm (see main text).

References

Ni, R. & Ouellette, N.T. On the tensile strength of insect swarms. *Phys. Biol.* **13**, 045002 (2016).

Reynolds, A.M. On the origin of the tensile strength of insect swarms. *Phys. Biol.* **16**, 046002 (2019).

IX: An illustrative example of a further application: modelling of honeybee drone congregation areas and flyways

Males of many insect species form dense, lek-like aerial swarms, above visual cues known as swarm markers. These often maintain a relatively stable size and shape even as individuals leave and others arrive, leading Sullivan [1981] to hypothesise that individual males move between adjacent swarms. Woodgate et al. [2021] provide the best evidence for a mating strategy in which individuals, honeybee drones, travel between multiple aerial leks whose locations are fixed. Woodgate et al. [2021] used harmonic radar technology [Riley et al. 1996] to record the flight paths of individual drones. They found clear evidence that drones favour certain areas in which to congregate, and that these areas (drone congregation areas, DCAs) were stable over two sequential years. Surprisingly, drones often visit multiple potential lekking sites within a single flight and take shared flight paths, 'flyways' between them. Flights between such sites, and between such sites and the hive are relatively straight. Once inside a drone congregation area, drones display convoluted, looping flight patterns those velocity and acceleration statistics closely resemble that of midges [Okubo 1986, Kelley and Ouellette 2013]. Drones thus use the same mechanisms for swarm cohesion as midges or mosquitos but on a far larger spatial scale (the congregations had a radius of approximately 50m, c.f. approximately 10cm for swarms of *Chironomus riparius* midges [Kelley and Ouellette 2013]).

Here I suggest that the flyways and the unidirectional flights between them are emergent properties of the DCAs rather than being distinctly different entities and with distinctly different behaviours. The suggestion stems from the results of numerical simulations for the trajectories of individuals in the presence of 2 DCAs. Model formulation directly mirrors that for swarming midges [Reynolds and Ouellette 2016].

Woodgate et al. [2021] reported that DCAs, like midge swarms [Okubo 1986, Kelley and Ouellette 2013] have to good approximation Gaussian density profiles. Consequently, the overall density profile for 2 DCAs can be represented by a bi-Gaussian distribution,

$$p(x) = \frac{1}{2}\frac{1}{\sqrt{2\pi}\sigma_x}\left(exp\left(-\frac{(x-\bar{x})^2}{2\sigma_x^2}\right) + exp\left(-\frac{(x+\bar{x})^2}{2\sigma_x^2}\right)\right) \quad \text{(S13)}$$

Woodgate et al. [2021] also reported that velocities within the DCA's are to good approximation Gaussian distribution. A 1-dimension stochastic trajectory simulation model that is consistent with a bi-Gaussian density profile and Gaussian velocity statistics is given in

$$du = -\frac{u}{T}dt + \sigma_u^2\frac{dlnp}{dx}dt + \sqrt{\frac{2}{T}}\sigma_u d\xi \quad \text{(S14)}$$

$dx = udt$

where *x* and *u* are the position and velocity of an individual at time *t*, *T* is a velocity correlation timescale and $d\xi(t)$ is an incremental Wiener process with correlation property $\overline{d\xi(t)d\xi(t+\tau)} = \delta(\tau)dt$ [Reynolds and Ouellette 2016, Supplementary Material V]. In accordance with observations [Woodgate et al. 2021] individuals located within the cores of the DCAs are effectively bound to the centre of the DCAs by a force (mean acceleration $\sigma_u^2 \frac{dlnp}{dx}$) that to good approximation grows linearly with distance from the centres. These effective restorative forces ensure that the positions of the simulated individuals are consistent with the prescribed density profile (Fig. S17a). In accordance with observations [Woodgate et al. 2021] the model predicts that convoluted flight patterns within the DCAs are interspersed with occasional unidirectional flights between the DCAs (typically along routes where the density profiles for individual DCAs overlap most strongly) that are is reminiscent of the observed unidirectional flight behaviours on the flyways (Fig. S17b,c). Similar results (Fig. S18) were obtained with 2-dimensional trajectory simulation models. This suggests that the flyways and the unidirectional flights along them are emergent properties of the DCAs as the flyways and directed flights do not enter the model formulation. This may explain why, as observed [Woodgate et al. 2021]: DCAs occur where flyways converge; almost nowhere in the vicinity of the DCAs is unvisited; and if the DCAs are stable then so are the flyways. The simple, minimal model may therefore have explanatory capacity, accounting for various observations of honeybee drones. Moreover, it can be readily extended. More elaborate modelling [Reynolds 2019, 2020] that takes account of intrinsic noise may, for example, explain why the DCAs appear to be in tension [Woodgate et al. 2021] and why DCAs, like some midge swarms [Poda et al. 2019], are not located directly over swarm markers [Woodgate et al. 2020].

One big question raised by the study of Woodgate et al. [2021] is why do drones switch between DCAs when lekking vertebrates are faithful to a single location? Although the switching between DCAs is here predicted to be accidental it may nonetheless be advantageous: either because when queens are rare in the environment, a regular patrol between multiple locations gives a better chance of finding a queen than staying in a single place; or because gatherings of drones have properties that are attractive to queens and that drones hunt around for a particularly good DCA to join. This suggests that there could be selection for *maintaining* switching rather than selection *for* switching. Nonetheless, switching is only predicted to occur when the DCAs are sufficiently close together. This is consistent

with Feugère et al. [2021] who reported that remote mosquito swarms are acoustically isolated.

Acknowledgements

I thank Joe Woodgate for stimulating communications about DCAs.

References

Feugère, L., Gibson, G., Manoukis, N.C. & Roux, O. Mosquito sound communication: are male swarms loud enough to attract females? *J. Roy. Soc. Int.* **18**, 20210121 (2021).

Okubo, A. Dynamical aspects of animal grouping: swarms, schools, flocks, and herds. *Adv. Biophys*. **22**, 1-94 (1986).

Kelley, D.H. & Ouellette, N.T. Emergent dynamics of laboratory insect swarms. *Sci. Rep.* **3**, 1073, 1-7 (2013).

Poda, S.B. et al. Sex aggregation and species segregation cues in swarming mosquitoes: role of ground visual markers. *Para. & Vects.* **12**, 589 (2019).

Reynolds, A.M. & Ouellette, N.T. Swarm dynamics may give rise to Lévy flights. *Sci. Rep.* **6**, 30515 (2016).

Reynolds, A.M. On the origin of the tensile strength of insect swarms. *Phys. Biol.* **16**, 046002 (2019).

Reynolds, A.M. Intrinsic stochasticity and the emergence of collective behaviours in insect swarms. *Euro. Phys. J. E.* **44**, 22 (2021).

Riley, J.R., Smith, A.D., Reynolds, D.R., Edwards, A.S., Osborne, J.L., Williams, I.H., Carreck, 359 N.L., & Poppy, G.M. Tracking bees with harmonic radar. *Nature* **379**, 29–30 (1996).

Sullivan, R.T. Insect swarming and mating. *Florida Entomol.* **64**, 44-65 (1981).

Woodgate, J.L., Makinson, J.C., Rossi, N., Lim, K.S. Reynolds, A.M., Rawlings, C.J. & Chittka, L. Harmonic radar tracking reveals that honeybee drones navigate between multiple aerial leks. **102499**, *iSci.* (2021).

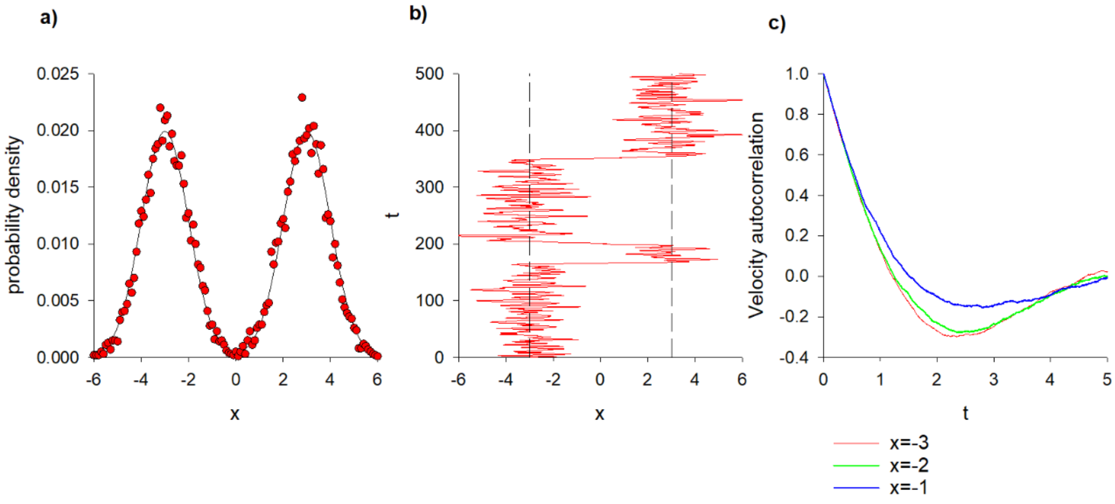

Figure S17a). Simulated trajectories are consistent with the presence of DCAs. Distribution of positions of simulated individuals (•) matches the prescribed bi-Gaussian density profile. **b) Simulated flight pattern as a function of time.** In accordance with observations [Woodgate et al. 2021] the model predicts that convoluted flight patterns within the DCAs are interspersed with occasional unidirectional flights between the DCAs that are reminiscent of the observed flight behaviours on the flyways. **c) Velocity autocorrelation functions** for trajectories starting from the centre a DCA (red line), within a DCA (green line) and between DCAs (blue line). Motions are underdamped (oscillatory) within the DCAs and nearly critically damped (unidirectional) between DCAs. Results are shown for $\bar{x} = 3$ and with all other model parameters set to unity.

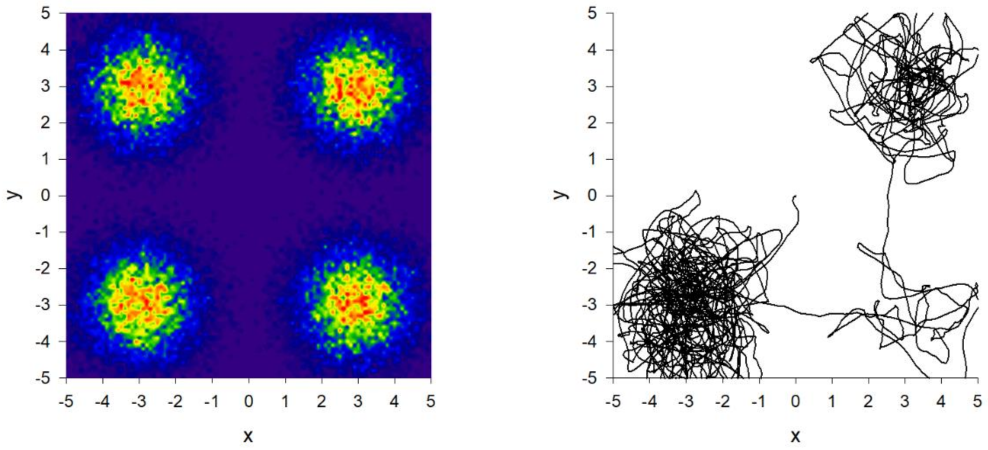

Figure S18. Example simulation data from a 2-dimensional stochastic model. The heat map (left) shows the locations of 4 prescribed DCA's. The trajectory (right) shows, as observed [Woodgate et al. 2021], convoluted flights within DCAs and unidirectional flights between DCAs.
